# TFEB promotes Ginkgetin-induced ferroptosis via TRIM25 mediated GPX4 lysosomal degradation in EGFR wide-type lung adenocarcinoma

**DOI:** 10.7150/thno.106469

**Published:** 2025-02-10

**Authors:** Hao-Jie Wang, Ling-feng Dong, Li-Li Ding, Xiu-Yuan Miao, Yu-Wen Zhang, Li-Ping Zhao, Li-Hua Yu, Zhen-Rong Guan, Ya-Ping Jiang, Xiao-Qi Tang, Ya-Xin Yan, Jian-Shu Lou

**Affiliations:** 1 School of Pharmacy, Hangzhou Normal University, Hangzhou, Zhejiang 311121, China.; 2 Key Laboratory of Elemene Class Anti-Cancer Chinese Medicines; Engineering Laboratory of Development and Application of Traditional Chinese Medicines; Collaborative Innovation Center of Traditional Chinese Medicines of Zhejiang Province, Hangzhou Normal University, Hangzhou, Zhejiang 311121, China.; 3 Shanghai Key Laboratory of Complex Prescription and MOE Key Laboratory for Standardization of Chinese Medicines, Institute of Chinese Materia Medica, Shanghai University of Traditional Chinese Medicine, Shanghai 201203, China.

**Keywords:** ginkgetin, ubiquitination, ferroptosis, LUAD, lysosome, TFEB

## Abstract

**Rationale**: TFEB activation is associated with prolonged survival in LUAD patients, suggesting potential benefits of TFEB agonists in LUAD treatment. In this study, we identify ginkgetin (GK), derived from *Ginkgo folium*, as a natural TFEB agonist, which has demonstrated promising anticancer effects in our previous research. TFEB activation has been shown to promote GPX4 degradation, inducing ferroptosis; however, the specific E3 ligases, deubiquitinating enzymes (DUBs), and types of polyubiquitination chains involved remain unclear. The unique mechanisms associated with natural compounds like GK may help elucidate the underlying biological processes. Here, we describe a novel biological event involved in the lysosomal degradation of GPX4 induced by TFEB activation through the utilization of GK.

**Methods**: TFEB activation was induced with GK, and TFEB knockout cells were generated using CRISPR-Cas9. The activity of TFEB and its relationship with ferroptosis were assessed by immunoprecipitation, labile iron pool and lysosomal activity assays. The types of polyubiquitination chains, E3 ligases, and DUBs involved in GPX4 degradation were analyzed using LC-MS, immunoprecipitation, and immunofluorescence. These findings were further validated in an orthotopic xenograft SCID mouse model.

**Results**: GK binds to and activates TFEB, leading to TFEB-mediated lysosomal activation and GPX4 degradation, which induces ferroptosis in LUAD cells. These effects were impaired in TFEB knockout cells. Mechanistically, K48-linked polyubiquitination of GPX4 was required for GK induced GPX4 lysosomal translocation. TFEB knockout reduced both K48-linked ubiquitination and lysosomal translocation of GPX4. Additionally, GK promotes the binding of TFEB and TRIM25. TRIM25 and USP5 were found to competitively bind to GPX4, with TFEB activation favoring TRIM25 binding to GPX4 and reducing the interaction of USP5 and GPX4. These findings were confirmed in a xenograft SCID mouse model using TFEB knockout LUAD cells.

**Conclusion**: This study identifies, for the first time, GK as a promising TFEB agonist for LUAD treatment. TFEB activation promotes TRIM25-mediated K48-linked polyubiquitination and lysosomal degradation of GPX4, driving ferroptosis. This ferroptosis-driven mechanism offers a novel strategy to enhance ferroptosis-based anti-LUAD therapies.

## Introduction

Lung cancer is the leading cause of cancer-related mortality worldwide 1. In China, the incidence of lung cancer ranks highest among all types of tumors, with a five-year survival rate below 20% 2, and effective long-term treatment options remain lacking. Among the types of lung cancer, non-small cell lung cancer (NSCLC) accounts for more than 80%. Early symptoms of lung cancer are not apparent, and over 65% of patients are diagnosed at an advanced stage 3. Lung adenocarcinoma (LUAD) is the most prevalent subtype of NSCLC, particularly common in women and non-smokers 4. Most patients with advanced-stage disease are not suitable candidates for surgery and require pharmacological treatment. Common pharmacological treatments include chemotherapy, targeted therapy, and immunotherapy. Chemotherapy is highly toxic and poorly tolerated by patients. Targeted therapy is effective only for specific genetic mutations and is prone to developing resistance, a significant portion of LUAD lacks targeted therapies, either due to the absence of key genetic mutations (e.g., EGFR wide-type) or the challenges in effectively targeting oncogenic mutations (e.g., KRAS mutations) 4,5. Immunotherapy has a low response rate, benefits a limited patient population, and can cause severe adverse effects such as myocarditis. Additionally, all these treatments—chemotherapy, targeted therapy, and immunotherapy—are associated with the risk of tumor hyperprogression, necessitating immediate discontinuation and a switch to alternative therapies 6. Consequently, there is an urgent need to identify more effective treatment modalities.

Ferroptosis is an iron-dependent form of cell death. The upregulation of the labile iron pool (LIP) and lipid peroxidation are the key features and primary mechanisms for inducing ferroptosis 7. Lung cancer cells exhibit an addiction to iron, often enhancing iron uptake to increase intracellular iron levels 8-9. In addition, lung cancer tissue exists in an environment with a higher concentration of oxygen than other tissues, necessitating the ability to withstand significant oxidative stress 10. This leads to the enhancement of antioxidant signaling pathways to cope with the elevated levels of reactive oxygen species (ROS) in lung cancer cells 11. This iron and antioxidant dependence renders lung cancer cells more vulnerable to ferroptosis, as the disruption of iron and redox homeostasis is likely to trigger ferroptosis. Targeting ferroptosis represents a promising strategy for the treatment of non-small cell lung cancer (NSCLC). Drugs capable of inducing ferroptosis hold significant value both as monotherapies and in combination therapies for NSCLC 8,12.

Recent studies indicated that lysosome, an organelle for degradation, is also a key hub for maintaining LIP availability and regulating ferroptosis. Lysosome receives extracellular iron via endocytosis, reducing it to Fe²⁺ for intracellular use, and facilitating its release into the cytoplasm to form LIP 13. Lysosome activation can mobilize iron and induce ferroptosis 14. In addition, the activation of lysosome can facilitate the degradation of ferroptosis negative regulators, which could also trigger ferroptosis. Furthermore, several studies showed that inhibition on lysosome attenuated erastin-induced ferroptosis 15. Thus, it was considered that ferroptosis is a lysosome cell death process, lysosome activation is an effective way to induce ferroptosis.

Given the essential role of lysosomes in inducing ferroptosis, there has been significant interest in developing pharmacological strategies that target lysosomes 15,16. TFEB is the master regulator for lysosome activity 17. Lysosome activation by TFEB is now considered to be a promising strategy for triggering ferroptosis. The research on the precise mechanism for TFEB regulating ferroptosis is still in its infancy. The regulation of TFEB in ferroptosis depends on the cell type. The inhibition effect of TFEB on ferroptosis was found in neuronal precursor cell PC12, this inhibition was triggered by TFEB mediated decrease on cellular LIP via upregulating on transferrin receptor 1(TfR1) and iron storage protein ferritin 18. Conversely, TFEB activation induced by natural compounds can trigger ferroptosis by degrading negative regulators of ferroptosis in cancer cells. For instance, several studies have demonstrated that TFEB promotes the degradation of ferritin to induce ferroptosis in cancer cells 19,20. In line with this, our previous study was the first to report that TFEB activation promotes GPX4 lysosomal degradation, triggering ferroptosis in NSCLC cells 21. However, the specific type of polyubiquitinated chain and the E3 enzyme involved in GPX4 lysosomal degradation are not yet fully understood.

GPX4 inhibition is the dominant mechanism for ferroptosis induction, rather than other fractional regulating signal axes, e.g., elevation of ROS, cellular iron levels, etc. 22,23. Recently, it was reported that TRIM25 mediated K48-linked polyubiquitination of GPX4 responsible for its proteasome degradation. In the past, it was considered that K48-linked polyubiquitin chain usually signal proteins to proteasomal degradation. However, it was reported that both K63- and K48- linked polyubiquitin chain target substrates to lysosomal degradation 24. As K48-linked polyubiquitination also involved in lysosomal degradation. It is intriguingly to figure out if TRIM25 mediated K48-linked polyubiquitination could signal GPX4 for lysosomal degradation, and if this process is regulated by TFEB. Moreover, ubiquitination and deubiquitination constitute counterbalancing processes in cells. DUBs function to antagonize the signals initiated by ubiquitin conjugating enzymes and ligases by removing ubiquitin from their substrates. GPX4 can be deubiquitinated by various ubiquitin-specific-processing proteases, including USP14, USP15, USP10, USP7, USP8, and USP25 25,26. Which DUB counter TRIM25 mediated K48-linked ubiquitination of GPX4 induced by TFEB activation has not yet been studied.

Studying the effect of TFEB activators on GPX4 lysosomal degradation may unveil novel biological events during this process, especially for some natural products, which usually have unique and unexplored mechanisms in anticancer activity. For instance, the promotion of TFEB on GPX4 lysosomal degradation is found with a natural TFEB activator β-ELE 21. Our previous study found a promising natural TFEB activator, ginkgetin (GK), a bioflavonoid uniquely found in Ginkgo biloba leaves. Additionally, we first reported that GK exhibits a promising anticancer effect in LUAD 27 and can enhance the anticancer effect of cisplatin by promoting ferroptosis at concentrations that achieve optimal synergy with cisplatin 28. However, the precise role of GK in ferroptosis and its relationship with TFEB and TRIM25 mediated lysosomal degradation has not been fully elucidated.

In this study, we illustrated the role of TFEB activation in TRIM25 mediated lysosomal degradation and the specific DUB involved in this process via a natural product GK, which could unveil the role of K48-linked ubiquitination induced by TRIM25 in lysosomal degradation, and the regulation of TFEB during this process. This unveiled mechanism offers a novel approach to lysosome activation-induced ferroptosis, further affirming its value in anti-lung cancer therapy.

## Methods

### Reagents and antibodies

GK was obtained from Chengdu Biopurify Phytochemicals Ltd. (BP0642, China, with a purity of ≥98%). GK was dissolved in DMSO (D8418, Sigma, USA, purity ≥99.9%). The antibodies used were as follows: GAPDH (60004-1), β-actin (66009-1), TRIM25 (12573-1), HSC70 (10654-1), USP5 (10473-1), HA (81290-1), Ubiquitin (10201-2), TFEB (13372-1) were purchased from Proteintech. PCNA (2586), solute carrier family 7 member 11 (SLC7A11) (12691), Phospho-TFEB (Ser122) (86843), Lamin B1 (13435), Ubiquitin (P4D1) (3936), FTH (4393), Phospho-TFEB (Ser211) (37681), 14-3-3 (8312), K63-linkage Specific Polyubiquitin (5621s), K48-linkage Specific Polyubiquitin (8081s) were purchased from Cell Signaling Technology. GPX4 (ab125066), TFEB (ab267351), Ferritin (ab75973), LAMP2A (ab18528), LAMP2 (ab25631) were purchased from Abcam. Flag (F1804), LAMP1 (sc-20011), GFP (sc-9996s) were purchased from Santa Cruz Biotechnology.

### SPR assay

The SPR assay was conducted using a GE Life Sciences Biacore S200 instrument equipped with a GE series S CM5 sensor chip. Initially, TFEB was covalently immobilized onto the chip surface via its amine groups, achieving an immobilization level of approximately 6500 RU. Various concentrations of GK (100 µM, 33.33 µM, 11.11 µM, 3.70 µM, 1.23 µM, 0.41 µM, 0.137 µM) were then passed over the chip surface, and their interactions with TFEB were monitored in real time. The entire assay was conducted in a buffer of 10 mM HEPES, pH 7.4, 150 mM NaCl, 0.05% P20, and 2% DMSO, at a constant temperature of 25 °C. Data analysis was performed using the GE Biacore S200 control software.

### MST assay

The MST measurement for the binding of GK to TFEB was performed using the High-Sensitive Microscale Thermophoresis Detection System (Monolith, NanoTemper Technologies, Germany). Cells were collected 48 h after transfection with pcDNA3.1-GFP or pcDNA3.1-TFEB-GFP plasmids, and cells were lysed by MST buffer (50 mM HEPES, 150 mM NaCl, 0.05% Tween-20, pH 7.8). GK at 30 mM was 2-fold diluted with DMSO to a final concentration of 3.662 μM. GK solutions at different concentrations were then further diluted 10-fold with PBST (137 mM NaCl, 2.5 mM KCl, 10 mM Na_2_HPO_4_, 2 mM KH_2_PO_4_, 0.05% Tween-20, pH 8.5). Subsequently, 10 μL of GK solution at different concentrations diluted with PBST was incubated with 10 μL of cell lysate. After 15 min incubation at room temperature, all the samples were loaded into MST standard glass capillaries and measurement was carried out at 40% MST power and 100% excitation power using the MO. Control software. Each experiment was performed in triplicate for all measurements. Data analysis was conducted using MO. Affinity Analysis software (NanoTemper Technologies). The dissociation constant (Kd) was calculated following the protocol provided by NanoTemper Technologies. The fluorescence change in the MST signal was normalized (Fnorm), where Fnorm is defined as Fhot/Fcold, with Fhot representing the fluorescence at 20 s post-IR laser heating and Fcold measured at 0 s. A dose-response curve was generated by plotting Fnorm against the ligand concentration. The Kd value for the interaction between TFEB and GK was derived from the saturation binding curve at equilibrium.

### Cell culture

The A549 cells were obtained from the American Type Culture Collection (ATCC, USA). The NCI-H460 and SPC-A-1 cells were obtained from the Cell Bank of the Chinese Academy of Sciences (Shanghai, China). NCI-H460 and SPC-A-1 cells were cultured in Roswell Park Memorial Institute (RPMI) 1640, and A549 cells were cultured in Ham's F-12 Kaighn's Modification Medium (MA-0230, Meilunbio, China), supplemented with 10% FBS and 100 units/mL Penicillin/Streptomycin, and the cells cultured at 37 °C with 5% CO_2_.

### Transfection

A549 cells were seeded at a density of 9 × 10⁵ cells per 10 cm culture dish and allowed to adhere overnight. The following day, each plasmid (8 µg) was diluted in 500 µL of jetPRIME® buffer (Polyplus, 101000046) and vortexed for 10 seconds. Next, 20 µL of jetPRIME® reagent was added to the mixture, vortexed for another 10 seconds, and incubated at room temperature for 10 min to facilitate the formation of transfection complexes. The transfection mixture (500 µL) was then added dropwise to the cells in serum containing medium. After 12 h of incubation, the medium was replaced with fresh medium, and the cells were cultured for an additional 12 h before being treated with GK.

### Western blot analysis

Treated cells were washed twice with 1× PBS and lysed in RIPA buffer (1 mM EGTA, 1 mM EDTA, 5 mM NaF, 1% Triton x-100, 1% NP-40, 10 μg/mL Aprotinin, 10 μg/mL Leupeptin, and 1 mM PMSF). Ultrasonic lysis of cells was performed, and the whole cell lysate protein concentration was quantified using a BCA Protein Assay Kit (FD2001, FD, China). The cell lysates were boiled in sample loading buffer at 95 °C for 5 min. Proteins were separated on 4-20% sodium dodecyl sulfate (SDS)-polyacrylamide gel electrophoresis (PAGE) and transferred to polyvinylidene difluoride (PVDF) membrane (Millipore, Bedford, MA, USA). The PVDF membrane was then blocked with 5% non-fat dry milk in 1× TBST for 1 h at room temperature. After blocking, the PVDF membrane was incubated with the primary antibody overnight at 4 °C. The next day, the membrane was incubated with the secondary antibody for 1 h at room temperature. The immunoreactivity signals were developed using ECL reagent, and immunoreactive protein bands were detected by the ChemiDoc™ Imaging System. The gray levels of protein bands were obtained using Image Lab software.

### Anti-GFP nanobeads preparation

NHS-activated agarose beads were washed with 1 mM HCl and equilibrated with 10 mM HBS (20 mM HEPES, pH 7.0, 150 mM NaCl) three times. The resin was incubated with nanobodies (1 mg nanobody : 100 μL resin) at 4 °C overnight. After the overnight incubation, the resin was washed with 10 column volumes of blocking buffer (0.1 M Tris-HCl, pH 8.0) and then incubated for 2 h at room temperature to deactivate any unreacted NHS sites. The resin was then washed with six cycles of wash buffer, alternating between buffer 1 (0.1 M Tris-HCl, pH 8.0, 0.5 M NaCl) and buffer 2 (0.1 M sodium acetate, 0.5 M NaCl, pH 4.0). Finally, the anti-GFP resin was equilibrated in storage buffer (10 mM Tris-HCl, pH 8.0, 150 mM NaCl, 20% ethanol) and stored at 4 °C. GFP nano-beads and their interaction with the target protein were measured by an immunoprecipitation assay.

### Bioinformatics analysis

The expression level of TFEB in normal lung tissue and LUAD tissues with different stages and pan-cancer analysis was analyzed by UALCAN analysis (http://ualcan.path.uab.edu). The Kaplan-Meier survival analysis was based on online websites GEPIA2 (http://gepia2.cancer-pku.cn/#index) and Kaplan-Meier Plotter (http://kmplot.com/analysis/). The relationship between TFEB and gene functional states was analyzed by CancerSEA (http://biocc.hrbmu.edu.cn/CancerSEA/). The following public databases were searched: the Cancer Genome Atlas (TCGA) database (https://gdac.broadinstitute.org/), Genotype-Tissue Expression (GTEx) dataset (https://gtexportal.org/home/datasets), CancerSEA database (http://biocc.hrbmu.edu.cn/CancerSEA/) and Kaplan-Meier Plotter database (http:// kmplot.com/analysis/).

### Immunoprecipitation

Cells were lysed in the IP lysis buffer (150 mM NaCl, 50 mM Tris-HCl pH 7.4, 1 mM EDTA, 1 mM EGTA, 0.5% NP-40, 5% glycerol, 1 mM PMSF, 5 mM NaF, 1 mM β-glycerophosphate, and 50 mM N-Ethylmaleimide). For ultrasonic lysis, the lysate was incubated with an antibody for 8 h at 4 °C. The immunocomplex was captured and incubated overnight with protein A/G (sc-2003, Santa). For proteins with a GFP tag, the lysate was incubated with 20 μL GFP-nanobeads overnight at 4 °C. GFP-nanobeads were washed three times using IP lysis buffer, and the immunocomplex was eluted from the beads by 2× SDS-PAGE sample buffer.

### Lipid peroxidation assay

Lipid peroxides were examined using BODIPY 581/591 C11 (#D3861, Invitrogen). A549 cells (7.5 × 10⁴) were seeded in 6-well plates and allowed to adhere overnight. After treatment with GK, the cells were washed twice with PBS and incubated with BODIPY 581/591 C11 (10 µM) in the dark for 30 min. Following incubation, the cells were washed twice with PBS, trypsinized for 1 min, and digestion was halted using medium containing 20% FBS. The cells were then collected by centrifugation at 800 × g for 6 min and resuspended in 1 mL PBS. The average fluorescence intensity of 1 × 10^4^ cells was measured using a flow cytometer (BD Biosciences, USA) with an excitation wavelength of 488 nm. Data were analyzed using FlowJo software. The mean fluorescence intensity (MFI) was determined by comparing fluorescence intensity values between treated and untreated samples, providing a quantitative measure of lipid peroxidation levels.

### Lysosome acidification measurement

Approximately 7.5 × 10^4^ cells were seeded in 6-well plates and incubated overnight. Cells were then treated or untreated with GK (15 μM) for 24 h. The cells were incubated with 50 nM Lyso-Tracker^®^ Red DND-99 for 30 min in the dark. After washing twice with PBS, trypsin was added and cells were collected. Then cells were analyzed by flow cytometry (BD Biosciences, USA) at 561 nm excitation. A total of 10,000 cells in each sample was analyzed, the data were analyzed using FlowJo software.

### Lysosomal activity assay

Magic Red was added to GK (15 μM) treated and untreated cells for 30 min in the dark, then cells were collected by trypsinization. The cells were analyzed by flow cytometry (BD Biosciences, USA) at 561 nm excitation. The intensity of fluorescence was positively correlated with the activity of cathepsin B. A total of 10,000 cells in each sample was analyzed, the data were analyzed using FlowJo.

### Cell viability assay

The cells were seeded onto 96-well plates. After treatment, the cell viability was detected by MTT or CCK-8 assay. For the MTT assay, the cells were exposed to 20 μL MTT (5 mg/mL) for 4 h at 37 °C in a 5% CO₂ incubator. The culture medium was then discarded, and 150 μL DMSO was added and shaken for 3 min. The absorbance at a wavelength of 562 nm was determined using a Multiskan™ FC Microplate Photometer (ThermoFisher scientific, USA). For the CCK-8 assay, after treatment with GK, 100 μL of culture medium containing a 10× concentration of 10 μL CCK-8 (HY-K0301, MCE) was added to the cells and incubated for 1 h. The absorbance at a wavelength of 450 nm was determined using a Multiskan™ FC Microplate Photometer (ThermoFisher Scientific, USA).

### Lysosome pH measurement

The cells were seeded in a 96-well plate at a density of 3×10³ per well. After attachment, the cells were treated with GK (15 μM) for 24 h, and then stained with LysoSensor™ Yellow/Blue DND-160 at the final concentration of 1 μM. The cells were then washed with 1× PBS, and the blue fluorescence (Ex/Em = 329 nm/440 nm) and yellow fluorescence (Ex/Em = 384 nm/540 nm) were detected. The higher value of yellow/blue fluorescence ratio indicates a more acidic environment in lysosome with lower pH value.

### Quantitative PCR with reverse transcription

Total RNA was extracted using RNA-easy isolation reagent (R701-01-AA, Vazyme, China). And cDNA was synthesized with Hiscript IV RT SuperMix (R4423-01, Vazyme, China). qRT-PCR was performed using ChemQ SYBR qPCR Mater Mix (Q311-02, Vazyme, China). The relative mRNA expression levels were analyzed using the 2^-ΔΔCT^ method. ACTB was used as a reference gene for mRNA. The following primers were used: 5′-TTCCCGGAGCTT TACTTTAACG' (S) and 5′-CAAGTCCTCTAGCGTCTCGC' (AS) for ATP6V0D1; 5′-GCCTTCCGACACCTCTTCC' (S) and 5′-CCACGGACATACGCATACCG' (AS) for MCOLN1; 5'-CTTCGACAACCTGATGCAGC'(S) and 5'-TACTTGGAGTCTGTGCCACC'-(AS) for CTSD; 5′-GAGGCAAGACCGAAGTAAACTAC' (S) and 5′-CCGAACTGGTTACACGGGAA' (AS) for GPX4; 5′-CATGTACGTTGCTA TCCAGGC' (S) and 5′-CTCCTTAATGTCACGCACGAT' (AS) for ACTB.

### Lentivirus production and infection

pLenti-based vectors for mammalian expression of 3×Flag-tagged GPX4 were transfected into HEK-293T cells with PMDLg/PRRE, pMD2.G, and PRSV-REV packaging vectors for 8 h using Lipofectamine 3000. The cell medium was then replaced with fresh medium containing no antibiotics. The culture supernatant was collected 48 h after transfection and centrifuged at 800 × g for 15 min at room temperature to remove cell debris. One volume of virus concentration solution (8.5% PEG 8000, 0.4 M NaCl) was added to three volumes of virus supernatant. After mixing, the mixture was rotated for 4 h at 4 °C at 60 rpm and then allowed to stand overnight. The solution was then centrifuged at 4 °C for 60 min at 1600 × g, and the supernatant was discarded. 300 μL of fresh F-12K medium containing suspended virus particles were added to the precipitate. The day before virus collection, A549 cells (2×10³) were seeded in a 96-well plate. The next day, the medium containing virus particles and 10 μg/mL polybrene was added to the 96-well plate, and the plate was centrifuged at 1600 × g for 60 min to increase transduction efficiency. After 24 h, the medium containing virus was removed and replaced with fresh F-12K medium for 72 h.

### Immunofluorescence staining

A549 cells were seeded on coverslips and treated with or without GK (15 μM) for 12 h or 24 h. The coverslips were washed with 1× PBS and fixed in 4% PFA for 15 min at room temperature, then blocked with blocking buffer (1× PBS, 5% BSA, 0.3% Triton™ X-100) for 1 h. Subsequently, cells were incubated with primary antibodies at 4 °C overnight and then incubated with Alexa Fluor 594-conjugated goat anti-mouse secondary antibody or Alexa Fluor 488-conjugated goat anti-rabbit secondary antibody for 1 h at room temperature. After incubation, the coverslips were mounted with ProLong Gold Antifade Reagent with DAPI (#8961, CST). Images were captured using a confocal laser-scanning microscope (Olympus Fluoview FV3000), and the co-localization coefficients were analyzed using Olympus Fluoview FV31S-DT software.

### LIP assay

Calcein-AM (CA-AM) and deferiprone (DFP) were used to detect the level of LIP. Calcein binds to Fe²⁺, and the fluorescence decreases. The binding of calcein to Fe is reversible. DFP binds to iron, causing the release of calcein. Finally, the fluorescence increases. Approximately 7.5 × 10⁴ cells were seeded in 6-well plates and incubated overnight. GK (15 μM) treated and untreated cells were incubated with 0.5 μM CA-AM for 15 min at 37 °C in the dark. The cells were washed twice with PBS and treated with or without DFP at a final concentration of 10 μM for 1 h in the dark. The cells were washed twice with PBS and trypsinized for 1 min. The digestion was halted by adding medium containing 20% FBS. The cells were collected, centrifuged at 800 × g for 6 min, and resuspended in 1 mL of PBS. Finally, the cell suspensions were analyzed using flow cytometry (BD Biosciences, USA) (λexc = 488 nm). Mean fluorescence intensity (MFI) increases with decreasing free iron content. The magnitude of iron chelation is calculated as ΔMFI (MFI CA-AM/DFP - MFI CA-AM alone) before and after treatment with GK. The level of LIP is positively correlated with the value of ΔMFI. A total of 10,000 cells in each sample was analyzed, the data were analyzed using FlowJo software.

### Orthotopic LUAD xenograft SCID mouse model

A549 and A549 TFEB^-/-^ cells were retrovirally transduced with a fusion protein reporter construct encoding GFP and firefly luciferase. The cells stably expressing the luciferase reporter gene were selected by puromycin screening. A549 WT luciferase cells and A549 TFEB^-/-^ luciferase cells (1 × 10⁶) were orthotopically implanted into the lungs of 5-6 week-old male SCID mice to establish the LUAD orthotopic xenograft SCID mouse model. Seven days post-implantation, the mice were sacrificed and randomly assigned into four groups: A549 WT control, A549 WT GK, A549 TFEB^-/-^ control, and A549 TFEB^-/-^ GK, with six mice per group, ensuring that each group had a similar mean luciferase-luciferin signal. GK solution (2% DMSO, 8% Cremophor EL, 90% NaCl) was administered intraperitoneally (120 mg/kg/day) the day after grouping. Mice were imaged *in vivo* every week to monitor changes in the luciferase-luciferin signal, which is directly proportional to tumor size. For imaging, mice were injected intraperitoneally with 50 μL of luciferin solution (30 mg/mL) per 10 g body weight and imaged 5 min post-injection using a Biospace Lab Photon Imager Optima (Nesles-la-Valee, France). Fluorescence signal intensity from the lungs was measured on days 0, 8, 15, and 21. Tumor inhibition rate was calculated based on the fluorescence intensity. On day 21, mice were sacrificed, and tumor tissues were either frozen at -80 °C or fixed in 4% PFA for immunofluorescence (IF) or immunohistochemistry (IHC), respectively. All animal procedures were performed in accordance with the National Research Council Guidelines for Laboratory Animal Care and Use and were approved by the Experimental Animal Ethics Committee of Hangzhou Normal University (Approval number: 2021-1125).

### Immunohistochemistry

Tumor samples were fixed in 4% paraformaldehyde, dehydrated with ethanol, cleared in xylene, and embedded in paraffin wax. Tissue blocks were sectioned at a thickness of 6 μm. The tissue sections were incubated overnight with proliferating cell nuclear antigen (PCNA) and then incubated with a secondary antibody for 1 h at room temperature. Immunohistochemistry (IHC) was performed to analyze the cellular distribution of PCNA. Images of four random visual fields were captured using a fluorescence microscope (DS-Ri1, Nikon, Japan). The mean IOD (Integrated Optical Density) of PCNA was analyzed using Image Pro Plus 6.0 software.

### Statistical analysis

The experimental data were based on three or more independent replicates. Statistical analysis was performed using GraphPad Prism 9.5 software. Data are presented as mean ± SD, and the significance between two groups was assessed using Student's t-test. One-way ANOVA was applied to compare differences among multiple groups, followed by Tukey's honest significant difference (HSD) test for pairwise comparisons. A P-value of < 0.05 was considered statistically significant; ns indicates no significant difference, * denotes *P* < 0.05, ** denotes *P* < 0.01, *** denotes *P* < 0.001, and **** denotes *P* < 0.0001.

## Results

### GK promotes TFEB-mediated lysosome activation

**Figure 1:**
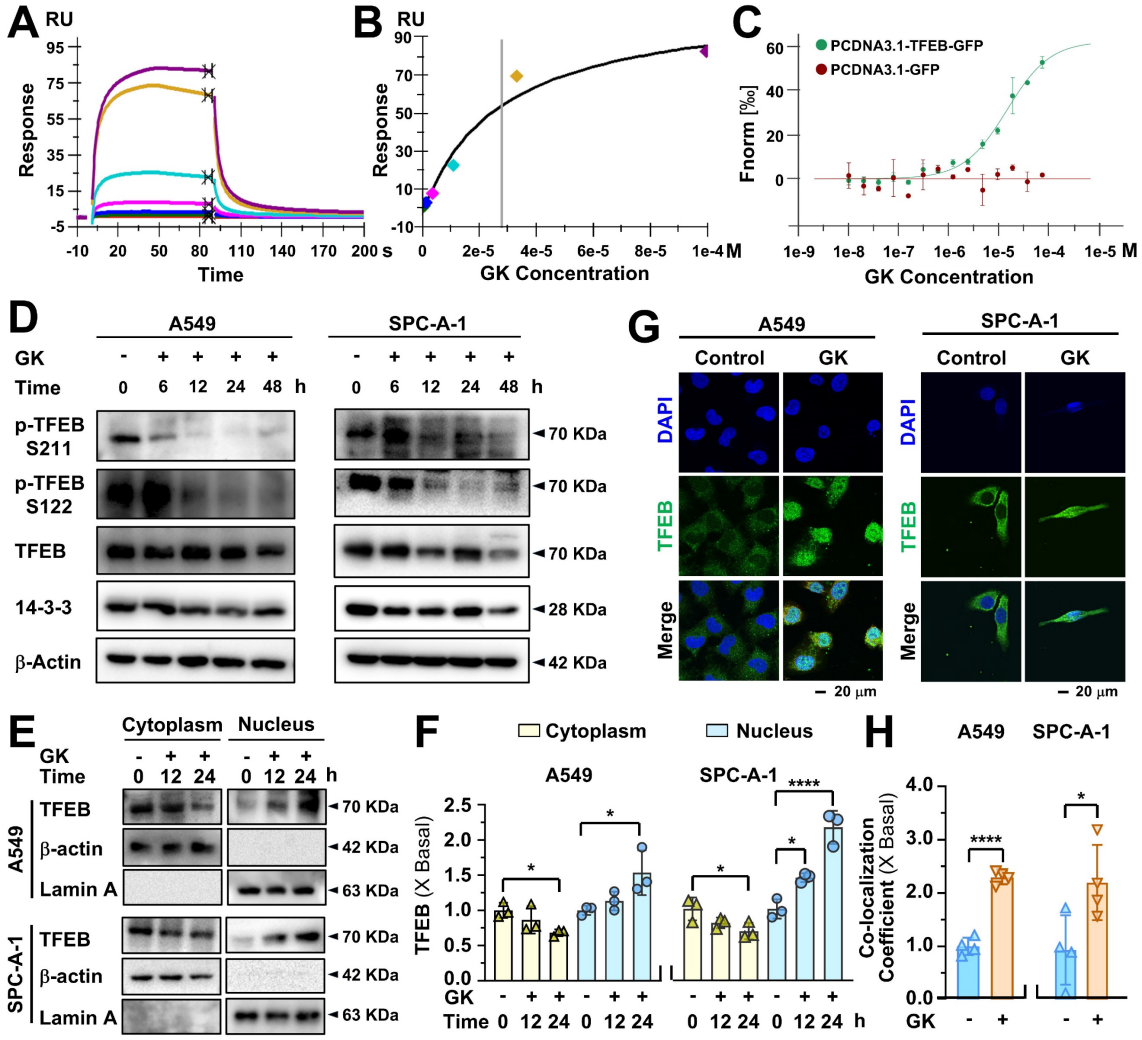
** GK binds to TFEB and induces TFEB activation.** (**A**) The binding affinity of GK to TFEB was determined by the SPR assay. TFEB protein was immobilized on a CM5 chip, then GK solution flowed over. The concentrations shown are ranging from 100 µM to 0.41 µM with three times dilution. (**B**) Data from (A) were fitted to the Langmuir equation, the dissociation constant (Kd) was determined using the steady-state model. (**C**) Cells were transfected with pcDNA3.1-GFP or pcDNA3.1-TFEB-GFP and subsequently lysed. The lysates were incubated with various concentrations of GK for 15 min. The binding affinity between TFEB and GK was measured using a High-Sensitivity Microscale Thermophoresis Detection System. The normalized binding curve of TFEB and GK is presented, with the binding curve yielding a Kd of 2.8 × 10^-5^ M. (**D**) LUAD cells (A549 and SPC-A-1) were treated with GK for 6, 12, 24, and 48 h. Western blot was conducted to analyze the protein levels of TFEB, p-TFEB (Ser122), p-TFEB (Ser211), and 14-3-3. (**E**) The cytoplasmic and nuclear protein of LUAD cells (A549 and SPC-A-1) were extracted after 24 h GK treatment. The protein expression of TFEB in each fraction were investigated by western blot. β-actin was served as the marker of cytoplasm, while Lamin A serves as the marker of nucleus. (**F**) The semi-quantitative analysis of TFEB protein expression in (E). *n* = 3, *P < 0.05, ****P < 0.0001. (**G**) LUAD cells (A549 and SPC-A-1) were treated with GK for 24 h, the nuclear translocation of TFEB was observed by immunofluorescence. Scale bar = 20 μM. (**H**) The co-localization was analyzed by Olympus Fluoview FV31S-DT software. Co-localization coefficients from (G) were calculated by measuring the co-localizing pixels between TFEB (green fluorescence) and DAPI (blue fluorescence) relative to the total number of pixels for the nuclei (DAPI channel). *n* = 4, *P < 0.05, ****P < 0.0001.

Our previous study demonstrated that the anticancer effect induced by GK can be sharply reversed by chloroquine (CQ), a lysosomal inhibitor, indicating that GK exerts its anticancer activity through lysosomal activation. TFEB is the master regulator of lysosome activation. Thus, we sought to determine if GK could activate TFEB and consequently lead to lysosome activation. Firstly, we observed whether GK can bind to TFEB. The surface plasmon resonance assay revealed that the sensorgram curve displayed a dose-dependent response (Figure 1A-B). The dissociation constant (Kd) value was determined using the steady-state model. Additionally, the MST assay further confirmed the binding affinity of GK to TFEB. The average Kd value is 2.8 × 10^-5^ M.

Next, we analyzed the role of TFEB in non-small cell lung cancer (NSCLC) using available databases. Given that the function of TFEB varies across different cancer types and even among subtypes of the same cancer, we focused on the major NSCLC subtype, LUAD, which has the most extensive data available in databases. A pan-cancer analysis of TFEB expression in tumors compared to normal tissue revealed a significant downregulation of TFEB in LUAD (Figure S1A-B). We next explored the potential clinicopathological implications of this altered expression. Kaplan-Meier survival analyses using data from TCGA and GTEx (Figure S1C), along with the KM Plotter database (Figure S1D), demonstrated that reduced TFEB expression was associated with poorer overall survival, suggesting that TFEB may serve as a protective factor in LUAD progression. Further functional analysis using the CancerSEA database indicated that epithelial-mesenchymal transition (EMT), proliferation, and DNA damage negatively correlated with TFEB expression in LUAD (Figure S1E). Additionally, transcriptional analysis performed through the UALCAN platform (Figure S1F) revealed that TFEB expression is negatively correlated with different stages of LUAD. In summary, these findings suggest that TFEB functions as a tumor suppressor gene in LUAD. Activating TFEB may be beneficial for LUAD therapy.

Thus, we sought to determine whether GK could activate TFEB in LUAD. TFEB activation depends on its dephosphorylation and dissociation from 14-3-3, allowing its translocation into the nucleus. Here, GK treatment led to a time-dependent decrease in the levels of p-TFEB (S211) and p-TFEB (S122) in two EGFR wild-type LUAD cell lines: A549 and SPC-A-1. Although no significant changes were observed in the total levels of TFEB (Figure 1D and Figure S2A), the binding between TFEB and 14-3-3 decreased in a time-dependent manner across these two LUAD cell lines (Figure S2B). The GK induced decrease in TFEB phosphorylation led us to further investigate its nuclear translocation. The immunofluorescence assay showed a marked increase in green pixels colocalized with DAPI in LAUD cell lines after 24 h of GK treatment, indicating a strong effect of GK on promoting TFEB nuclear translocation (Figure 1G-H). To further validate this, we analyzed the nuclear and cytoplasmic fractions of these LAUD cells to observe changes in TFEB distribution. TFEB levels showed a time-dependent increase in the nuclear fraction and a corresponding decrease in the cytoplasmic fraction, with significant changes observed after 24 h, further confirming the promoting effect of GK on TFEB nuclear translocation (Figure 1E-F). We also observed the effect of GK on TFEB in another NSCLC subtype, large cell lung carcinoma (LCC), for which limited information regarding its relationship with TFEB is available in databases. Similarly, GK induced activation of TFEB was also observed in LCC, characterized by a decrease in p-TFEB levels (Figure S3A), reduced binding between TFEB and 14-3-3 (Figure S3B), and enhanced nuclear translocation of TFEB (Figure S3C-D). Collectively, these findings demonstrate that GK can bind to and activate TFEB in NSCLC cells.

**Figure 2:**
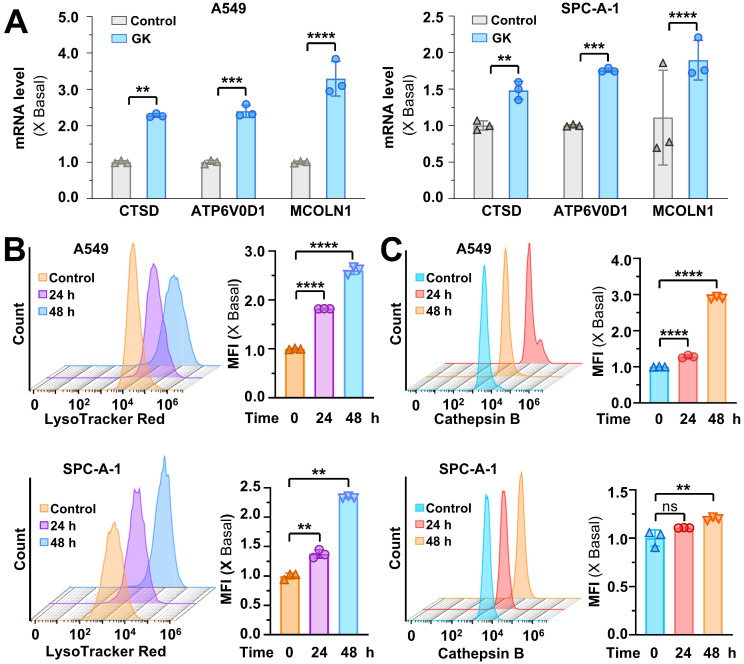
** GK promotes lysosome activation. (A)** LUAD cells (A549 and SPC-A-1) were treated with GK (15 μM) for 24 h. Following treatment, cells were harvested, and mRNA was extracted and subsequently reverse transcribed into cDNA. The mRNA level of CTSD, ATP6V0D1 and MCOLN1 were detected by qPCR. **(B)** LUAD cells (A549 and SPC-A-1) were treated with GK for 24, 48 h, then labeled with LysoTracker™ Red DND-99 (50 nM) for 30 min. Fluorescence intensity of 10,000 cells per sample was measured by flow cytometry. The fluorescence intensity of the cells was displayed in histograms (left panel), and the relative changes in mean fluorescence intensity (MFI) compared to the control group was quantified (right panel).** (C)** LUAD cells (A549 and SPC-A-1) were treated same as in (B), then stained with Magic Red for 30 min. The level of cathepsin B was analyzed by flow cytometry. Fluorescence intensity of 10,000 cells per sample was analyzed. The fluorescence intensity of the cells was displayed in histograms (left panel), and the relative changes in mean fluorescence intensity (MFI) compared to the control group was quantified (right panel). *n* = 3, **P < 0.01, ***P < 0.001, ****P < 0.0001.

The activation of TFEB can transcriptionally upregulate lysosomal genes, leading to lysosomal activation. Given the TFEB activation induced by GK, we sought to determine whether lysosomal gene expression was increased in GK treated NSCLC cells. Genes involved in lysosomal hydrolases (CTSD), lysosome membrane integrity (MCOLN1), and lysosomal acidification (ATP6V0D1) were all significantly upregulated in the LUAD (Figure 2A) and LCC cells (Figure S4A). This upregulation of genes related to lysosomal biogenesis contributed to lysosomal activation. To further assess this, we examined lysosomal activity in GK treated NSCLC cells using LysoTracker™ Red DND-99 and detected by flow cytometry. The mean fluorescence intensity (MFI) was significantly elevated in a time-dependent manner, confirming the activation effect of GK on lysosomes (Figure 2B and Figure S4B-C). In addition, we detected the changes of lysosome pH in GK treated LUAD cells via LysoSensor™ Yellow/Blue DND-160, the lysosome pH values were also significantly decreased both in A549 and SPC-A-1 cells characterized by the increased ratio of yellow/blue fluorescence, indicating more acidic environment in lysosome (Figure S4E-F). To corroborate these observations, we analyzed cathepsin B, a key protease involved in maintaining lysosome population and size. Similarly, mean fluorescence intensity significantly increased after 48 h of GK treatment in all three NSCLC cell lines (Figure 2C and Figure S4D). However, no significant changes were observed in NCI-H460 and SPC-A-1 cells after 24 h of GK treatment, with notable changes only occurring in A549 cells (Figure 2C and Figure S4D). These findings collectively indicate that GK activates lysosomes in LUAD and LCC cells, with the effect being more pronounced in A549 cells. Therefore, we selected A549 cells for further mechanistic studies.

**Figure 3:**
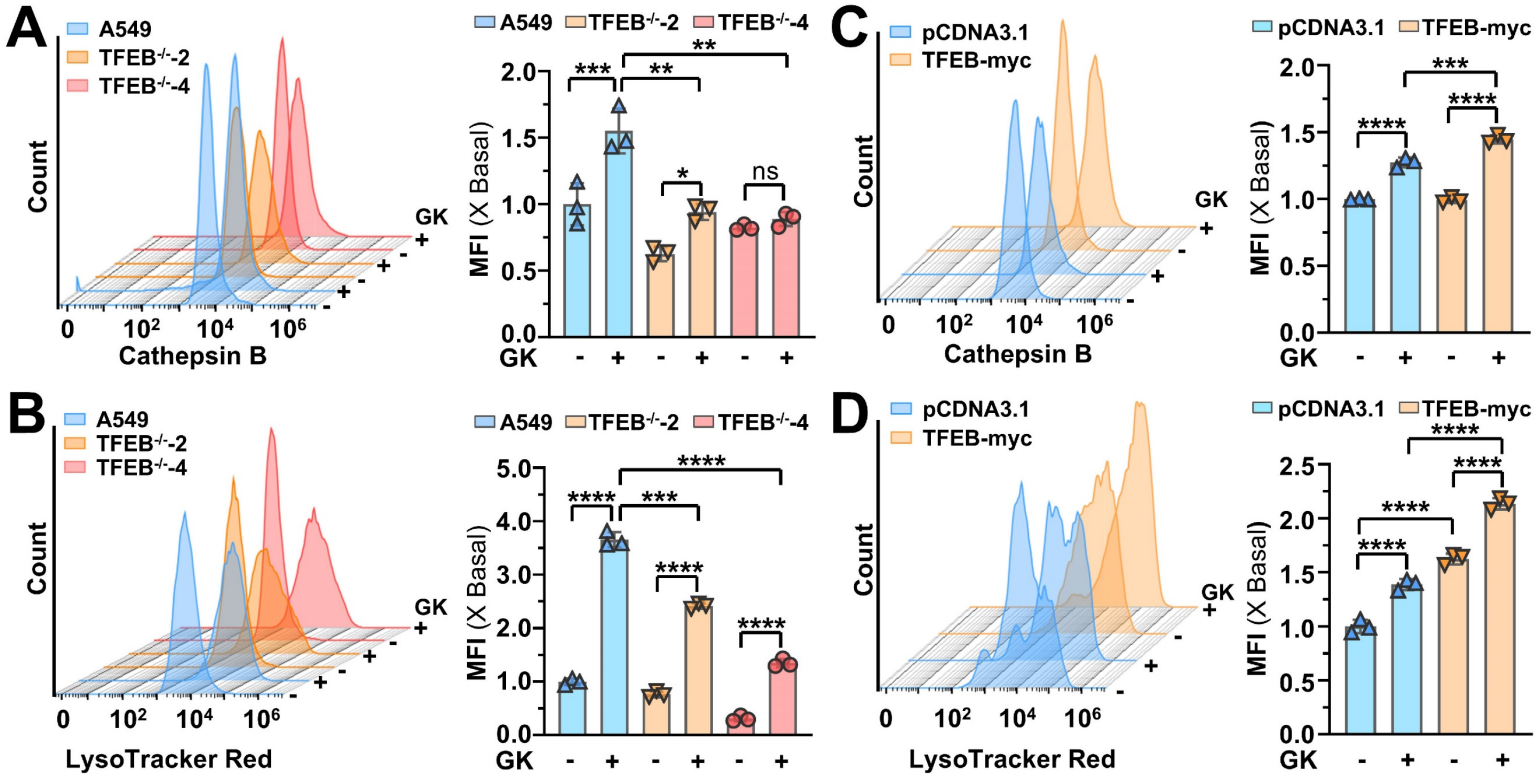
** TFEB is positively related to GK induced lysosome activation. (A)** A549 WT cells and TFEB knockout cells (TFEB^-/-^-2, TFEB^-/-^-4) were treated with GK for 24 h, then stained with Magic Red for 30 min. The level of cathepsin B was analyzed by flow cytometry. Fluorescence intensity of 10,000 cells per sample was analyzed. **(B)** Cells were treated same as in (A). Cells was labeled with LysoTracker™ Red DND-99 (50 nM) for 30 min. Fluorescence intensity of 10,000 cells per sample was measured by flow cytometry. **(C)** A549 cells were transfected with TFEB or mock-transfected with pcDNA3.1, then treated with GK for 24 h. The measurement of cathepsin B levels was performed as described in (A). **(D)** The transfection and GK treatment were performed as described in (C), and the detection of lysosomal activity was conducted as described in (B). The fluorescence intensity of the cells was displayed in histograms (left panel), and the relative changes in mean fluorescence intensity (MFI) compared to the control group was quantified (right panel). *n* = 3, **P < 0.01, ***P < 0.001, ****P < 0.0001.

In order to observe the role of TFEB in GK induced lysosome activation, we established TFEB knockout stable cell line to observed the changes on lysosome activation in GK treated LUAD cells (Figure S6A). The GK induced increase in lysosomal activity and cathepsin B levels were significantly attenuated by TFEB knockout (Figure 3A-B), while these effects were enhanced by TFEB overexpression (Figure 3C-D). Similarly, TFEB knockout compromised the decrease on lysosome pH in GK treated LUAD cells (Figure S4E). Lysosomes can be activated through either TFEB-dependent or TFEB-independent pathways. These results indicate that GK induces lysosome activation via a TFEB-dependent mechanism.

### GK promotes GPX4 lysosomal degradation by K48-linked ubiquitination

**Figure 4:**
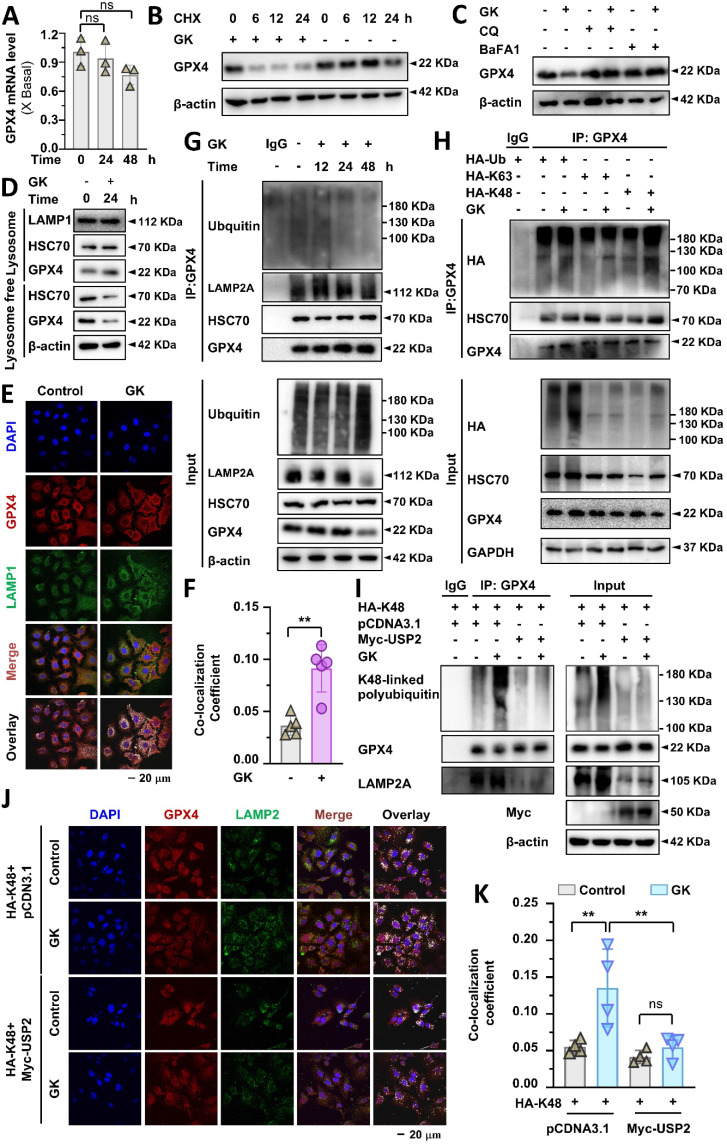
** GK promotes GPX4 lysosomal degradation and K48-linked ubiquitination. (A)** A549 cells were treated with GK (15 μM) for 24, 48 h. The cells were harvested, and mRNA was extracted and reverse transcribed into cDNA. The mRNA level of GPX4 was investigated by qPCR. *n* = 3.** (B)** A549 cells were treated with CHX (1 μg/mL), CHX+GK (1 μg/mL + 15 μM) for 6, 12, 24 h, the protein expression of GPX4 was observed by western blot. **(C)** A549 cells were treated with GK (15 μM) in the absence or presence of CQ (20 μM), BaFA1(160 nM) for 24 h. The protein expression of GPX4 was observed by western blot.** (D)** A549 cells were treated with GK (15 μM) for 24 h. The cells were harvest and the lysosome fraction was extracted by lysosome isolation kit. The protein expression of HSC70, GPX4 in both lysosome and lysosome free fractions were detected by western blot. LAMP1 serves as lysosome marker, and β-actin was serves as a marker for lysosome free fraction. **(E)** A549 cells were treated as described in (D), and the co-localization of GPX4 and LAMP1 was observed by immunofluorescence. Scale bar = 20 µM. **(F)** The co-localization was analyzed by Olympus Fluoview FV31S-DT software. The co-localization coefficient was determined from (E).* n* = 5, **P < 0.01. **(G)** A549 cells were treated with GK for 12, 24, 48 h, cells were collected and lysed. 100 μg of the cell lysates of each sample were subdivided and used as input control. The left cell lysates were subjected to immunoprecipitation via protein G beads and GPX4 antibody. Immunoprecipitated protein complexes and input were analyzed by western blot using GPX4, ubiquitin, LAMP2A, and HSC70 antibodies.** (H)** A549 cells were transfected with HA-Ub, HA-K63 or HA-K48, then each transcription group was treated with GK for 12 h. Then cells were harvest and lysed. 100 μg of the cell lysates of each sample were subdivided and used as input control. The left cell lysates were subjected to immunoprecipitation via protein G beads and GPX4 antibody. Immunoprecipitated protein complexes and input were analyzed by western blot using GPX4, HSC70, and HA antibodies.** (I)** A549 cells were co-transfected with HA-K48 and Myc-USP2, or HA-K48 and pcDNA3.1. Subsequently, each transcription group was either treated or untreated with GK for 12 h. The cells were collected and lysed, then performed immunoprecipitation assay as in (G). Immunoprecipitated protein complexes and input were analyzed by western blot using GPX4, LAMP2A, K48-linkage specific polyubiquitin and Myc antibodies.** (J)** The transfection and drug treatment were same as in (I)**.** The co-localization of LAMP2 and GPX4 were observed via immunofluorescence. Scale bar = 20 µM.** (K)** The co-localization was analyzed by Olympus Fluoview FV31S-DT software. The co-localization coefficient from (J) was calculated by determining the number of colocalized pixels of GPX4 (red fluorescence) with LAMP2 (green fluorescence) relative to the total number of LAMP2 pixels. Scale bar = 20 μM. *n* = 4, **P < 0.01.

In our previous study, we demonstrated that TFEB could promote GPX4 lysosomal degradation, and GK downregulates the protein level of GPX4^21^. In this study, we discovered that GK binds to and activates TFEB. Therefore, it became compelling to investigate whether GK induced TFEB activation promotes GPX4 lysosomal degradation and to explore any unknown biological events involved in this process. Firstly, we observed if GK transcriptionally regulated the level of GPX4. Fluorescence quantitative PCR assay revealed that no obvious changes of GPX4 mRNA level were observed in GK treated A549 cells (Figure 4A), which was also observed in another LUAD cells SPC-A-1 cells (Figure S5A), indicating GK has no effect on GPX4 transcription. Thus, the decline of GPX4 might be due to protein degradation. To validate this, we applied protein synthesis inhibitor CHX to observe the change on protein level of GPX4. When protein synthesis blocked by CHX, the GPX4 protein level were sharply decreased after GK treated for 6 h (Figure 4B and Figure S6C). However, in our previous study no obvious decrease on GPX4 was observed in 6h. Similarly, in GK treated SPC-A-1 cells, no obvious changes on protein level of GPX4 were observed in 12 h, while that was sharply decreased at the presence of CHX (Figure S5B). These results indicate that GK promoted GPX4 degradation. Next, to investigate whether GK could promote lysosomal degradation, we applied lysosomal degradation inhibitors chloroquine (CQ) and BaFA1 to examine the changes in GK induced GPX4 decline. Since GK significantly decreased GPX4 levels after 24 h of treatment, we selected this time point for observation. The GK induced decline in GPX4 was completely abolished by CQ and BaFA1(Figure 4C and Figure S6D), but not by the proteasome inhibitor MG132 (Figure S6B), indicating that GK may promote GPX4 lysosomal degradation.

Lysosomal degradation occurs after protein translocate to lysosome. Next, we want to figure out if the lysosome translocation of GPX4 is increased in GK treated A549 cells. In the lysosomal fraction, the amount of GPX4 was significantly increased after GK application, while it was decreased in the lysosome free fraction (Figure 4D and Figure S6E). In addition, GK increased the binding of GPX4 and lysosomal protein LAMP2A, which was also observed in SPC-A-1 cells (Figure 4G and Figure S5C). These data indicated an enhanced lysosomal translocation of GPX4, which was further supported by immunofluorescence assays showing a notable increase in co-localization of GPX4 with the lysosomal marker LAMP1 after GK treatment (Figure 4E-F). Intriguingly, the expression of HSC70, a cochaperone that selectively recognizes GPX4 to deliver it to the lysosome, was slightly increased in the lysosomal fraction and decreased in the lysosome free fraction. However, no obvious increase was observed in the binding of GPX4 and HSC70. Alternatively, a notable increase in the ubiquitination of GPX4 was observed after 12 h of GK treatment. This increase was not observed at 24 h and even decreased at 48 h (Figure 4G and Figure S6F). These data indicate that GK induced ubiquitination of GPX4 might facilitate its translocation to the lysosome. As the duration of GK treatment increased, GPX4 was progressively degraded in lysosomes, while the ubiquitination level of GPX4 concomitantly decreased.

The specific type of ubiquitin chain, such as K48-linked and K63-linked polyubiquitin chains, can signal proteins for localization to lysosomes for degradation 24. To identify the type of polyubiquitin chain responsible for GPX4 ubiquitination, we transfected HA-Ub-WT, HA-Ub-K48, and HA-Ub-K63 to observe changes in K48-linked and K63-linked ubiquitination of GPX4. A significant increase in K48-linked ubiquitination of GPX4 was observed in GK treated LUAD cells; however, no change was noted in K63-linked ubiquitination (Figure 4H and Figure S6G). The increased K48-linked polyubiquitination of GPX4 was also observed in SPC-A-1 cells (Figure S5C). These findings indicate that GK induced polyubiquitination of GPX4 is primarily K48-linked, which may signal GPX4 for lysosomal degradation. To observed the role of K48-linked ubiquitination in the lysosomal translocation, we use USP2 to specific remove K48-linked ubiquitination to investigate the change on GPX4 lysosomal localization. The removal of K48-linked ubiquitination abolished the GPX4 lysosomal localization, characterized by the undetectable increase in the co-localization of GPX4 and LAMP2A in USP2 overexpression LUAD cells (Figure 4 J-K), which was further confirmed by immunoprecipitation assay that the GK induced increase in the binding of GPX4 and LAMP2A was abolished by USP2-mediated removal of the K48-linked ubiquitination chain (Figure 4I). This phenomenon demonstrate that K48-linked ubiquitination is required for GPX4 lysosomal localization.

### GK-induced K48-linked polyubiquitination-mediated GPX4 lysosomal degradation was dependent on TFEB

**Figure 5:**
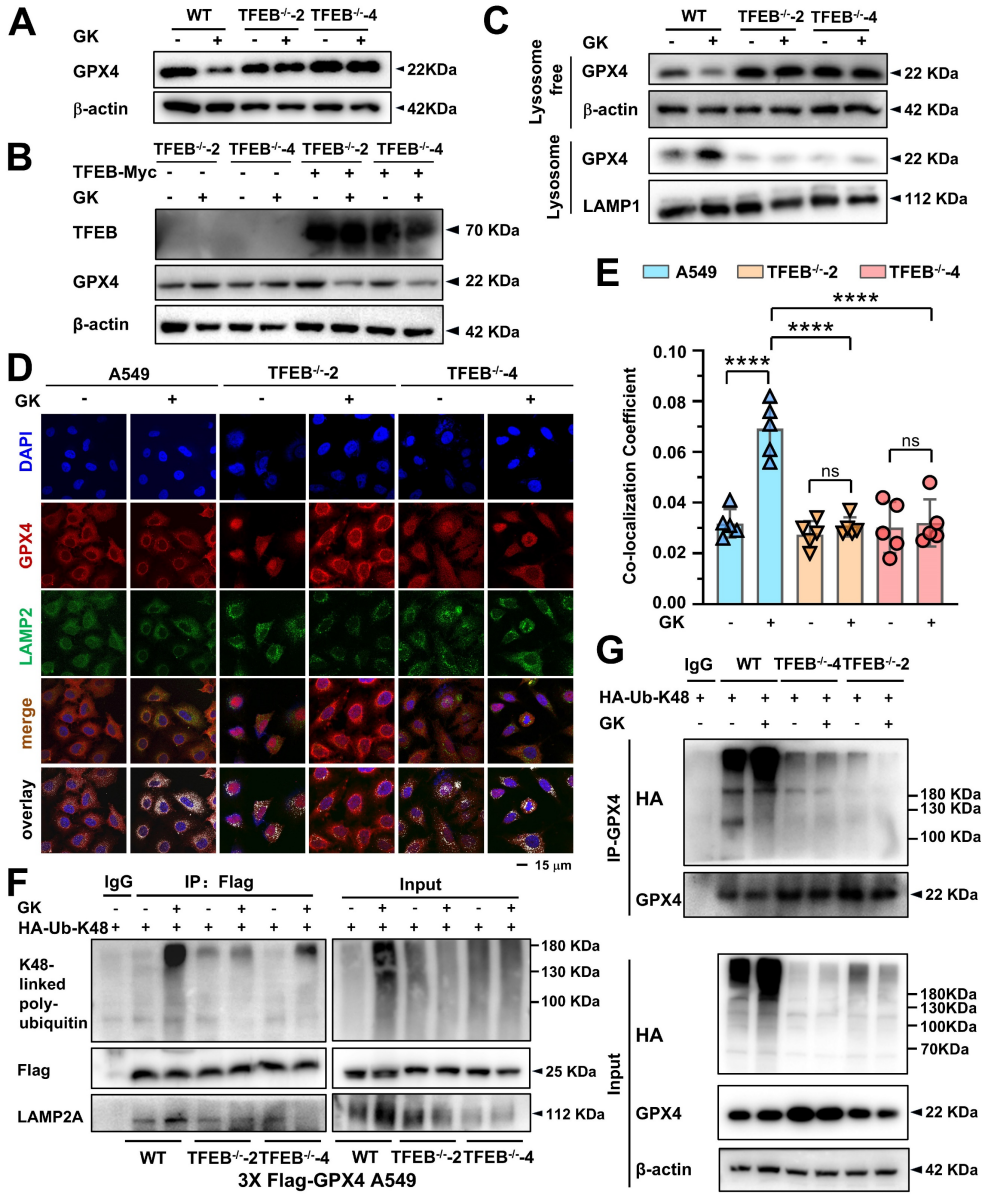
** TFEB promotes GK induced GPX4 lysosomal degradation. (A)** A549 cells and TFEB knockout A549 cells (TFEB^-/-^-2, TFEB^-/-^-4) were treated with GK (15 μM) for 24 h. The protein expression of GPX4 was determined by western blot.** (B)** TFEB knockout cells (TFEB^-/-^-2, TFEB^-/-^-4) were transfected with TFEB-Myc or mock transfected with pcDNA3.1, followed by a 24 h treatment with GK. The protein levels of TFEB and GPX4 were then analyzed by western blot. **(C)** A549 cells and TFEB knockout A549 cells (TFEB^-/-^-2, TFEB^-/-^-4) were treated with GK (15 μM) for 24 h. The cells were harvested, and lysosomes were extracted using a lysosome isolation kit. The protein level of GPX4, both in the lysosomal and lysosome free fractions, was investigated by western blotting. LAMP1 serves as a lysosomal marker, and β-actin serves as a marker for the lysosome free fraction. **(D)** A549 cells and TFEB knockout A549 cells (TFEB^-/-^-2, TFEB^-/-^-4) were treated with GK for 12 h, the co-localization of GPX4 and LAMP2 was observed via confocal microscopy. **(E)** The co-localization was analyzed by Olympus Fluoview FV31S-DT software. Co-localization coefficient was calculated by the colocalized pixels of GPX4 (red fluorescence) and LAMP2 (green fluorescence) relative to the total pixels of LAMP2. Scale bar = 15 μm. *n* = 5, ****P < 0.0001.** (F)** Flag-GPX4 stably transfected cells were transfected with HA-K48, then treated with GK (15 μM) for 12 h. 100 μg of the cell lysates of each sample were subdivided and used as input control. The left cell lysates were harvested and lysed, then performed immunoprecipitation via Flag antibody and protein G beads. The expression of K48-linked polyubiquitin, flag-GPX4 and LAMP2A in immunoprecipitants and input were investigated by western blot. **(G)** A549 cells and TFEB knockout A549 cells (TFEB^-/-^-2, TFEB^-/-^-4) were transfected with HA-K48, then treated with GK (15 μM) for 12 h. The left cell lysates were harvested and lysed, then performed immunoprecipitation via GPX4 antibody and protein G beads. The expression of K48-linked polyubiquitin, GPX4 in immunoprecipitants and input were investigated by western blot.

Our previous study demonstrated that TFEB activation is responsible for GPX4 lysosomal degradation; however, the detailed biological events involved in this process remain unclear. In this study, both TFEB activation and GPX4 lysosomal degradation were observed in GK treated LUAD cells, raising the intriguing possibility that GK induced TFEB activation drives GPX4 lysosomal degradation. Moreover, identifying the specific type of polyubiquitin chain and E3 enzyme involved in this process, which were not previously elucidated in TFEB regulation of GPX4 lysosomal degradation, remains a key focus. Here, we applied GK to both TFEB wild-type and knockout LUAD cells. GPX4 expression significantly decreased in the TFEB wild-type LUAD cells, while this reduction was completely abolished in the TFEB knockout LUAD cells (Figure 5A and Figure S7A). Conversely, when TFEB was overexpressed in the knockout cells, the reduction in GPX4 levels was restored (Figure 5B and Figure S7B). These findings indicate that TFEB is negatively correlated with GPX4 expression in GK treated LUAD cells.

Next, we investigated the role of TFEB in GK induced GPX4 lysosomal translocation and K48-linked ubiquitination. We first analyzed the levels of GPX4 in both the lysosomal and lysosome free fractions in wild-type and knockout LUAD cells. The GK induced increase of GPX4 in the lysosomal fraction and the corresponding decrease in the lysosome free fraction were completely abolished by TFEB knockout (Figure 5C and Figure S7C). This observation was further supported by immunofluorescence assays, where GK induced co-localization of GPX4 with LAMP2 was not significantly increased in TFEB stable knockout cells (Figure 5D-E). These results underscore the critical role of TFEB in facilitating GPX4 lysosomal translocation. Similarly, in GPX4 stable transfection and TFEB wild-type cells, GK markedly induced K48-linked ubiquitination of GPX4, an effect that was significantly diminished in TFEB knockout cells (Figure 5F-G and Figure S7D-E). Collectively, these findings demonstrate that TFEB promotes GK induced GPX4 lysosomal degradation.

### TRIM25 and USP5 competitively bind to GPX4, and GK promotes GPX4-TRIM25 binding in a TFEB-dependent manner

**Figure 6:**
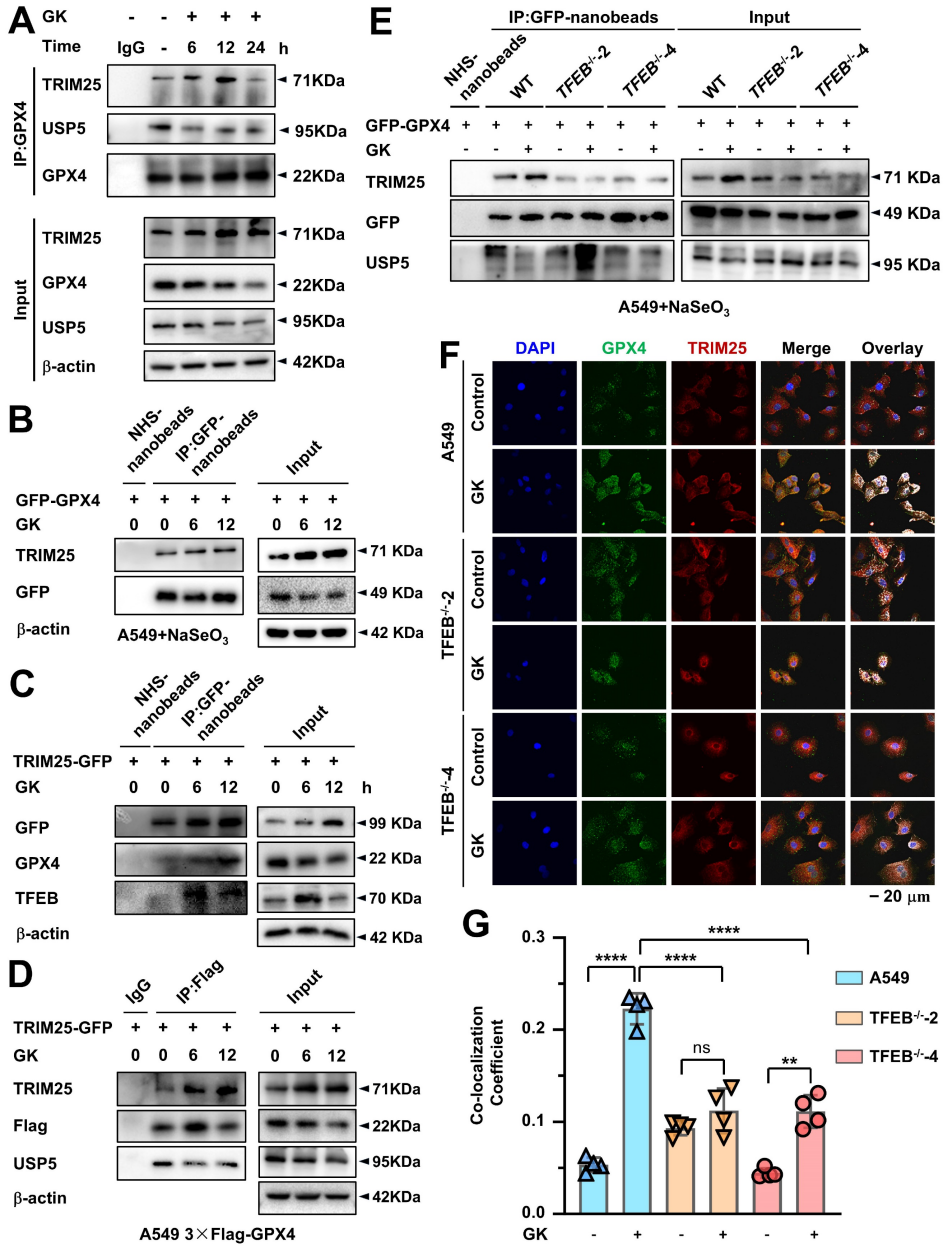
** TFEB promotes GK induced binding of GPX4 and TRIM25. (A)** A549 cells were treated with GK (15 μM) for 6, 12, and 24 h. After treatment, the cells were harvested and lysed. A portion (100 μg) of each cell lysate was used as input control. The remaining lysates were subjected to immunoprecipitation using protein G beads and GPX4 antibody. Both the immunoprecipitated fractions and input controls were analyzed by western blot with GPX4, TRIM25, and USP5 antibodies. **(B)** A549 cells were transfected with GFP-GPX4 and supplemented with NaSeO₃ (1 μM). The cells were treated with GK for 6, 12 h, then collected and lysed. A portion (100 μg) of each cell lysate was used as input control, while the remaining lysates were subjected to immunoprecipitation using GFP-nanobeads. The protein levels of GFP-GPX4 and TRIM25 in the immunoprecipitated fractions and input controls were analyzed by western blot. **(C)** A549 cells were transfected with TRIM25-GFP and treated with GK for 6, 12 h. Immunoprecipitation was performed as described in (B). The immunoprecipitated fractions and input controls were analyzed by western blot using GFP, GPX4, and TFEB antibodies. **(D)** TRIM25-GFP was transfected into A549 cells stably expressing 3×Flag-GPX4. Cell treatments were performed as described in (B). Immunoprecipitation was conducted as outlined in (C). The immunoprecipitated fractions and input controls were analyzed by western blot using TRIM25, Flag, and USP5 antibodies. **(E)** A549 cells and TFEB knockout A549 cells (TFEB^-/-^-2, TFEB^-/-^-4) were transfected with GFP-GPX4. Each transfection group was treated with GK (15 μM) for 12 h. Immunoprecipitation was performed as described in (B). The immunoprecipitated fractions and input controls were analyzed by western blot using TRIM25, GFP, and USP5 antibodies. **(F)** A549 cells and TFEB knockout A549 cells (TFEB^-/-^-2, TFEB^-/-^-4) were treated GK (15 μM) for 12 h. The co-localization of GPX4 and TRIM25 was observed by immunofluorescence assay. **(G)** The co-localization of GPX4 and TRIM25 was analyzed using Olympus Fluoview FV31S-DT software. Co-localization was calculated based on the number of colocalized pixels of GPX4 (green fluorescence) and TRIM25 (red fluorescence) relative to the total number of pixels for GPX4 (green fluorescence). Scale bar = 20 μM. *n* = 4, **P < 0.01, ****P < 0.0001.

Given that E3 ubiquitin ligases facilitate the transfer of ubiquitin onto substrate proteins, we employed immunoprecipitation coupled with mass spectrometry (IP-MS) to identify the key E3 ligases responsible for GK induced lysosomal degradation of GPX4. In LUAD cells, IP-MS analysis of GPX4-interacting proteins revealed that the E3 enzyme TRIM25 was significantly increased following GK treatment (Figure S8A). Recently, it was reported that TRIM25 enhances K48-linked polyubiquitination of GPX4 29. Therefore, we hypothesized that GK may promote TRIM25-mediated K48-linked polyubiquitination of GPX4, triggering its lysosomal degradation. To investigate this, we first examined the changes in the binding of TRIM25 to GPX4 following GK treatment using endogenous immunoprecipitation. The binding between GPX4 and TRIM25 increased after 6 h of GK treatment, even though GPX4 did not show a decrease in expression at that time (Figure 6A). However, as shown in Figure 4B, GPX4 degradation became evident from 6 h of GK treatment. The binding between GPX4 and TRIM25 became more pronounced after 12 h of GK treatment, which coincided with significant GPX4 degradation and a slight decrease in its expression (Figure 4B, Figure S6C, Figure 6A and Figure S8B). Notably, the increase in GPX4-TRIM25 binding was no longer observed after 24 h of GK treatment, likely due to the substantial decrease in GPX4 levels at that time (Figure 6A and Figure S8B). The GPX4-TRIM25 binding was further confirmed through exogenous immunoprecipitation. GK treatment increased the binding of TRIM25 and GPX4 in A549 cells overexpressing with either TRIM25 or GPX4 alone, as well as in A549 cells overexpressing with both TRIM25 and GPX4 (Figure 6B-D and Figure S8C-E). Additionally, the increased interaction of GPX4 and TRIM25 was also found in GK treated SPC-A-1 cells (Figure S5C). As TFEB can promote GK induced GPX4 degradation, we want to figure out if TFEB is also critical for the GK induced binding of TRIM25 and GPX4. In GFP-GPX4 transfected cells, GK enhanced the binding of TRIM25 and GPX4, an effect that was completely abolished in TFEB knockout cells (Figure 6E and Figure S8F). This phenomenon was further corroborated by immunofluorescence assay, which showed increased co-localization of GPX4 and TRIM25 following GK treatment, with this increase significantly attenuated in TFEB knockout LUAD cells, both in A549 and SPC-A-1 (Figure 6F-G and Figure S5D).

Intriguingly, in IP-MS data, we also noticed that the deubiquitinating enzymes (DUBs) USP5 was decreased in GK treated LUAD cells (Figure S8A). Thus, we simultaneously observed the change on the interaction of GPX4 and USP5 after GK treatment. We found that with the increase in the interaction between GPX4 and TRIM25, there was a corresponding decrease in the interaction between GPX4 and USP5. These phenomena were observed in both endogenous and exogenous immunoprecipitation assays (Figure 6A, Figure 6D, Figure S8B, and Figure S8E). Moreover, in TFEB knockout cells, along with the abolition of GK induced binding between GPX4 and TRIM25, the GK induced decrease in the binding of GPX4 and USP5 was also attenuated, as demonstrated by immunoprecipitation (IP) (Figure 6E and Figure S8F). As ubiquitination and deubiquitination are counterbalancing processes, these results suggest that GK activates TFEB, which in turn inhibits GPX4 from binding to USP5 while promoting its interaction with TRIM25. This shift ultimately leads to K48-linked ubiquitination of GPX4 and its subsequent lysosomal degradation.

### GK induced ferroptosis was compromised by TFEB knockout

**Figure 7:**
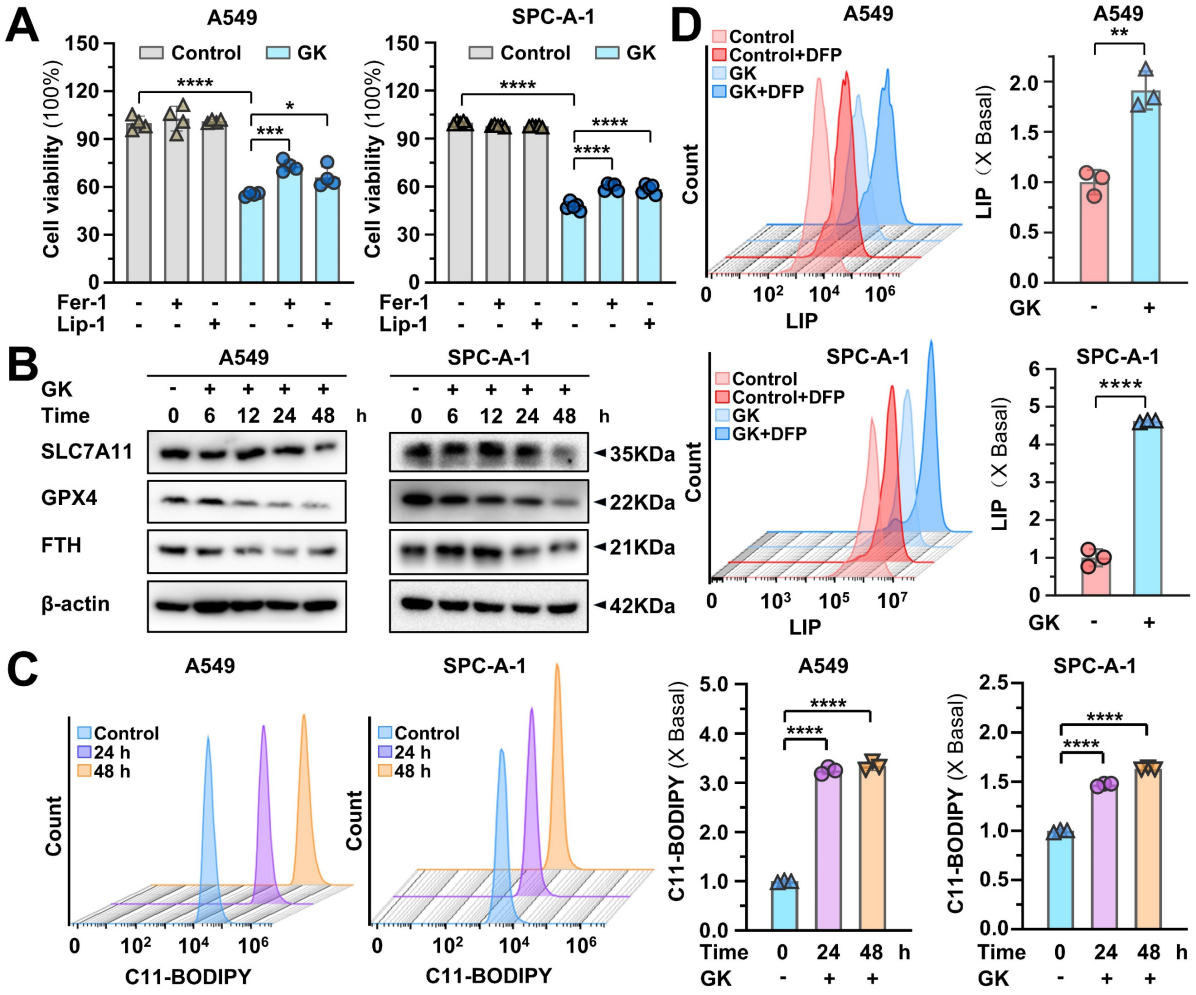
** GK promotes ferroptosis in LUAD cells. (A)** LUAD cells (A549 and SPC-A-1) were treated with GK (15 μM) for 48 h in the presence and absence of Ferrostatin-1 or liproxstatin-1. The proliferation inhibition of the cells was observed by MTT assay. *n* = 4, *P < 0.05, ***P < 0.001, ****P < 0.0001.** (B)** LUAD cells (A549 and SPC-A-1) were treated with GK (15 μM) for 6, 12, 24, 48 h. The protein levels of SLC7A11, GPX4, and FTH were observed by western blot. β-actin served as internal control. **(C-D)** LUAD cells (A549 and SPC-A-1) were treated with GK (15 μM) for 24, 48 h. The cells were collected and stained with BODIPY™ 581/591 C11 (10 μM) for 30 min. The level of lipid peroxidation was observed by flow cytometry (λexc =488 nm) (C). LUAD cells (A549 and SPC-A-1) were treated with GK (15 μM) for 24 h, CA-AM was added to cells at the final concentration of 0.5 µM, followed by adding iron chelator deferiprone (DFP, 100 μM) for 1 h or left untreated. The level of LIP was detected by flow cytometry (λexc=488 nm) (D). 10,000 cells for each sample were analyzed. Left panel: The fluorescence intensity of the cells was displayed in histograms. Right panel: Relative changes in mean fluorescence intensity (MFI) (C) or ΔMFI (D) compared to the control group. *n* = 3, **P < 0.01, ****P < 0.0001.

GPX4 is the hub for ferroptosis, its reduction can trigger ferroptosis. Our previous study demonstrated that the low concentration of GK combine with chemotherapy drug could promote ferroptosis 28. Thus, the regulation on TFEB mediated GPX4 lysosomal degradation waved us to figure out the impact of GK on ferroptosis. Firstly, we applied ferroptosis inhibitors to observe the changes on GK induced proliferation inhibition. The application of ferroptosis inhibitor Fer-1, Lip-1 significantly reversed the GK induced inhibition of LUAD cell (Figure 7A), which could not be reversed by the pyroptosis inhibitor LDC7559, the cuproptosis inhibitor TEPA, or the necrosis inhibitor Necrostatin-1 (Figure S9A). Our previous study also showed that the anticancer effect of GK could not be reversed by apoptosis inhibitors 30. In addition, the attenuation of ferroptosis inhibitors on GK induced inhibition of proliferation was also observed in LCC cells (Figure S10B). Thus, ferroptosis is the main cell death type triggered by GK. Furthermore, while the ferroptosis negative regulators SLC7A11 and FTH were slightly reduced in the two LUAD cell lines, the most pronounced decrease was observed in GPX4 (Figure 7B and Figure S10A), which was also observed in LCC cells (Figure S10C-D), indicating that GPX4 might be the key hub for GK induced ferroptosis in LUAD cells.

Next, we observed the two key characteristics in ferroptosis: lipid peroxidation and LIP. GK notably increase the level of lipid peroxidation in a time-dependent manner (Figure 7C). Similarly, LIP level was significantly increased after 24 h GK treatment in two LUAD cells (Figure 7D). Additionally, the elevation on LIP and lipid peroxidation were also observed in GK treated LCC cells (Figure S10E-F). To observe the key role of GPX4 in GK induced ferroptosis, we overexpressed GPX4 in LUAD cells to observe if the GK induced ferroptosis would be compromised. As we expected, GK induced inhibition on cell proliferation and the promotion on lipid peroxidation and LIP were all attenuated after GPX4 overexpression (Figure S9B-D). These data collectively suggest that GK promotes ferroptosis in LUAD and LCC cells, with the sharp decrease in GPX4 levels underscoring its pivotal role in GK induced ferroptosis.

**Figure 8:**
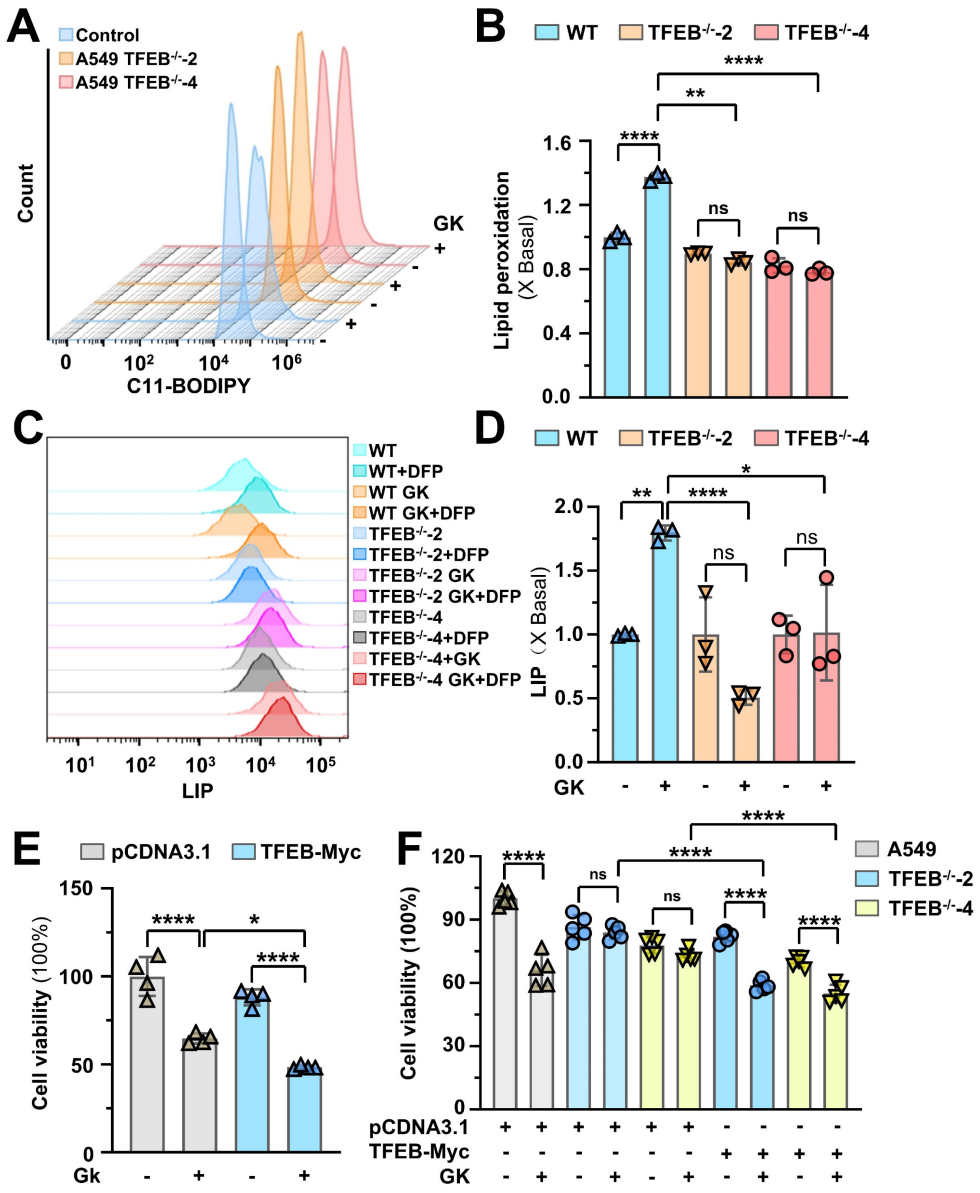
** GK induced ferroptosis compromised by TFEB knockout. (A)** A549 cells and TFEB knockout A549 cells (TFEB^-/-^-2, TFEB^-/-^-4) were treated with GK (15 μM) for 24 h. The cells were collected and stained with BODIPY™ 581/591 C11 (10 μM) for 30 min. The level of lipid peroxidation was observed by flow cytometry (λexc = 488 nm), 10,000 cells for each sample were analyzed. Histograms represents the fluorescence intensity of the cells. **(B)** The relative changes in mean fluorescence intensity (MFI) for each treatment group compared to the control group, quantified from (A).* n* = 3, **P < 0.01, ****P < 0.0001. **(C)** A549 cells and TFEB knockout A549 cells (TFEB^-/-^-2, TFEB^-/-^-4) were treated with GK (15 μM) for 24 h. Then the cells were stained with CA-AM (0.5 µM), followed by iron chelator deferiprone (DFP, 100 μM) for 1 h or left untreated. The level of LIP was detected by flow cytometry (λexc = 488 nm), 10,000 cells for each sample were analyzed. Histograms represents the fluorescence intensity of the cells. **(D)** The relative LIP level of each treated sample compared to the WT control group calculated from (C).* n* = 3, *P < 0.05, **P < 0.01, ****P < 0.0001.** (E)** A549 cells were transfected with TFEB-Myc or mock transfected with pcDNA3.1, then treated with GK (15 μM) for 48 h. The proliferation inhibition of the cells was observed by MTT assay. *n* = 4, *P < 0.05, ****P < 0.0001.** (F)** A549 cells were mock transfected with pcDNA3.1, TFEB knockout A549 cells (TFEB^-/-^-2, TFEB^-/-^-4) were transfected with TFEB-Myc or mock transfected with pcDNA3.1. Then each transfection group was treated with GK (15 μM) for 48 h. The proliferation inhibition of the cells was observed by MTT assay. *n* = 5, ****P < 0.0001.

To investigate the role of TFEB in GK induced ferroptosis, we applied GK treatment to both wild-type LUAD cells and TFEB knockout cells. The GK induced increases in lipid peroxidation and LIP levels were significantly attenuated in TFEB knockout cells (Figure 8A-D). Additionally, the GK induced inhibition of cell proliferation was enhanced by TFEB overexpression and reversed in TFEB knockout cells (Figure 8E-F). Notably, the inhibitory effect on proliferation was restored when TFEB was re-expressed in TFEB knockout cells (Figure 8F). These findings suggest that TFEB plays a crucial role in mediating GK induced ferroptosis.

### GK induced anticancer effect was attenuated by TFEB knockout in orthotopic SCID mice model

**Figure 9:**
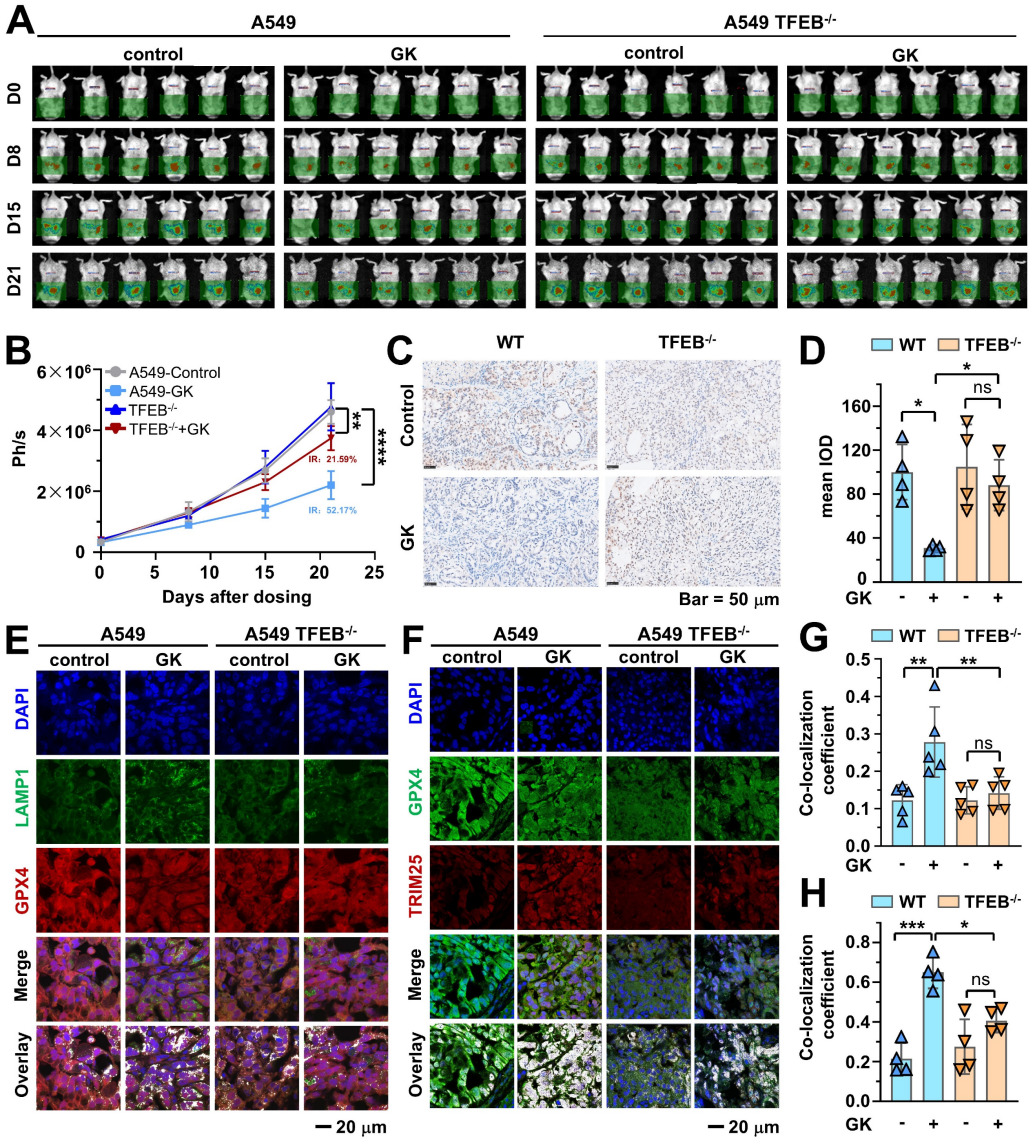
** TFEB knockout compromised GK induced anticancer effect.** A549-luci or A549^TFEB KO^-luci cells were injected into the right lung of male NOD/SCID mice. Five days post-implantation, the mice were randomized into four groups, each comprising six mice. The mice implanted with A549-luci or A549 TFEB KO-luci cells were administered GK (120 mg/kg) for 21 days. Following treatment, the mice were euthanized, and tumor tissue samples were either snap-frozen at -80 °C or fixed in 4% paraformaldehyde (PFA) for subsequent immunofluorescence (IF) or immunohistochemistry (IHC) analysis. Experimental procedures were conducted as detailed in the Materials and Methods section. **(A)** Representative bioluminescence images were captured at specified time points (Day 0, Day 8, Day 15, and Day 21) following GK administration. **(B)** Bioluminescence intensity in mice, expressed in radiance (Ph/s), with *n* = 6, **P < 0.01, ****P < 0.0001.** (C)** Immunohistochemical staining analysis showing PCNA-positive cells stained brown and nuclei stained blue. Representative images from each group are presented. **(D)** Each tumor tissue section was randomly chosen to determine the mean Integrated Optical Density (IOD) value of the positively stained region using Image Pro Plus software, reflecting PCNA expression levels. Scale bar = 50 μm. *n* = 4. *P < 0.05.** (E)** The co-localization of GPX4 and LAMP1 in tumor tissues was assessed using immunofluorescence. Scale bar = 20 μm. **(F)** Co-localization of GPX4 and TRIM25 in tumor was detected by immunofluorescence. Scale bar = 20 μm. **(G)** Co-localization coefficients from (E) were calculated by Olympus Fluoview FV31S-DT software. Co-localization coefficient was calculated by the colocalized pixels of GPX4 (red fluorescence) and LAMP1 (green fluorescence) relative to the total pixels of LAMP1. *n* = 5. **P < 0.01.** (H)** Co-localization coefficients from (F) were calculated by Olympus Fluoview FV31S-DT software. Co-localization coefficient was calculated based on the number of colocalized pixels of GPX4 (green fluorescence) and TRIM25 (red fluorescence) relative to the total number of pixels for GPX4 (green fluorescence). *n* = 4. *P < 0.05, ***P < 0.001.

To further investigate the role of TFEB in GK induced anticancer effect, we established orthotopic SCID mice model using A549-luci cells or A549 TFEB^-/-^-luci cells that stably express luciferase. In mice transplanted with A549-luci cells, the tumor burden, monitored through luciferase-luciferin signal, gradually increased during the first week, with rapid tumor growth observed from day 7. In contrast, mice treated with GK exhibited significantly slower tumor growth. By day 21, the inhibition rate in the GK treated group reached 52.17%. However, in mice transplanted with A549 TFEB^-/-^-luci cells, the GK induced anticancer effect was not as obviously as that in mice transplanted with A549-luci cells, as indicated by relatively faster tumor growth and a much lower inhibition rate of 21.59% (Figure 9A-B). These phenomena indicate that the absence of TFEB in tumor largely attenuated the anticancer effect of GK, which was further demonstrated with the expression of PCNA observed by IHC, GK notably decreased the expression of PCNA in A549-luci mice, while this decrease was less pronounced in A549 TFEB^-/-^-luci mice (Figure 9C-D).

To further examine the role of TFEB in regulating GPX4, we performed immunohistofluorescence to analyze the expression of GPX4, TRIM25, and the co-localization of GPX4 with both LAMP1 and TRIM25. In tumors from A549-luci mice, GK treatment led to a decline in GPX4 expression and an increase in the co-localization of GPX4 with LAMP1. However, these changes were not significant in A549 TFEB^-/-^-luci mice (Figure 9E and Figure 9G), indicating that the absence of TFEB attenuates GK induced decreases in GPX4 and increases in GPX4-TRIM25 interaction. Moreover, GK administration elevated the expression of TRIM25 and its interaction with GPX4 in tumors from A549-luci mice, an effect that was not evident in A549 TFEB^-/-^-luci mice (Figure 9F and Figure 9H). These findings suggest that TFEB positively regulates the GK induced increase in TRIM25 and its co-localization with GPX4.

## Discussion

Since the discovery of ferroptosis a decade ago, research advancements have underscored the increasingly significant role of lysosomal activation in the induction of ferroptosis. Inhibition of lysosomal function has been shown to impede ferroptosis 20, prompting the consideration of ferroptosis as a lysosomal cell death process 31. TFEB functions as a key regulator in the induction of ferroptosis. Upon activation, it promotes ferroptosis by degrading crucial negative regulators of ferroptosis. Our previous study demonstrated that suppressing lysosome activity sharply compromised GK induced anticancer effect. In addition, GK could promote ferroptosis in cisplatin treated LUAD cells. Recently, we were the first to report that drug-induced activation of TFEB can promote GPX4 degradation, thereby triggering ferroptosis. In our screening system, GK, a natural compound, can bind to TFEB and exhibits a promising effect on TFEB activation. Thus, elucidating the mechanism by which TFEB mediates the degradation of GPX4, facilitated by the natural TFEB activator GK, could uncover distinctive and specific biological events occurring during this process. Here, we've found that: (i) GK promoted K48-linked polyubiquitination of GPX4 and its lysosomal degradation, which was positively related to TFEB; (ii) K48-linked polyubiquitination is required for GK induced GPX4 lysosomal translocation. (iii) GK promoted the binding of TFEB and TRIM25. TRIM25 and USP5 competitively bind to GPX4, with GK induced TFEB activation promoting the binding of GPX4 to TRIM25 while inhibiting its binding to USP5; (iv) GK induced ferroptosis is dependent on TFEB activation; (v)TFEB knockout compromised the GK induced anticancer effect in an orthotopic xenograft SCID mouse model, as well as the lysosomal translocation of GPX4 and its interaction with TRIM25.

TFEB agonists have been successfully applied in various disease models 32, including spinal cord injury 33, kidney disease 34 and hyperoxia-induced bronchopulmonary dysplasia 35. The accelerated growth of cancer cells results in a significantly higher demand for energy and biosynthetic materials compared to normal cells 36. As a result, elevated lysosomal system activity is commonly observed in tumor cells, making TFEB an attractive target for cancer treatment. While some preclinical studies highlight potential negative effects of TFEB in cancer, it is important to note that its regulatory mechanism is not consistent across all cancer types, and may vary even between subtypes of the same cancer. Furthermore, clinical data support the beneficial role of TFEB in cancer treatment. For instance, data from the Human Protein Atlas demonstrate that low TFEB expression is associated with poor survival rates in renal and pancreatic carcinomas. Our analysis of the TCGA database similarly revealed that in LUAD, TFEB expression is lower in tumor tissues compared to normal tissues, and low TFEB expression correlates with poor survival outcomes. Additionally, recent studies have reported that TFEB overexpression enhances chemotherapy sensitivity 37 and promotes ferroptosis in NSCLC 21. Thus, TFEB agonists have potential benefits for LUAD, particularly through triggering ferroptosis.

Recently, accumulating evidence has highlighted the positive role of TFEB agonists in promoting ferroptosis. For example, quercetin and luteolin activate TFEB, enhancing lysosomal degradation of ferritin and subsequently increasing iron release to induce ferroptosis in breast and prostate cancer cells, respectively 19, 38. In line with this, inhibition of TFEB-mediated lysosomal degradation of ferritin by a TFEB inhibitor suppressed ferroptosis in osteosarcoma cells 39. In addition, the increased iron level also achieved by TFEB induced lysosomal membrane permeabilization (LMP) 40. At present, the majority of studies report that TFEB mediates ferroptosis induction by releasing iron from the lysosome, a key hub for cellular iron homeostasis, either through the degradation of ferritin or by disrupting LMP. However, research on the regulation of TFEB in ferroptosis is still in its early stages. Recently, through the pharmacological activation of TFEB, we discovered that TFEB-mediated GPX4 lysosomal degradation contributes to the induction of ferroptosis 21, providing new insights into the regulatory mechanisms of TFEB in ferroptosis. Since the action of natural compounds frequently reveals distinctive and novel mechanisms in anticancer research, it is offering to investigate the specific biological events related to GPX4 degradation in NSCLC cells following treatment with natural TFEB activators.

TFEB activators are commonly found in natural compounds. In our screening system, we identified GK, a unique compound derived from *Ginkgo Folium*, has binding affinity to TFEB. It has been reported that the TFEB inhibitor EO binds to TFEB at the region spanning Helix 1-loop-Helix 2, creating steric hindrance that obstructs its interaction with TFEB 41. In contrast, GK binds to TFEB at the interface between the HLH and LZ regions, which does not induce steric hindrance for TFEB dimerization or its binding to DNA. Further observations demonstrate the promising effect of GK on TFEB activation. This is evidenced by a significant increase in TFEB nuclear translocation, lysosomal gene expression in GK treated EGFR wild-type LUAD cells, known for their poor response to targeted therapies. Additionally, GK induced GPX4 lysosomal degradation and ferroptosis were also observed in these LUAD cells. Therefore, elucidating regulators such as specific types of ubiquitination chains, E3 ligases, and DUBs will help to illuminate the novel mechanisms underlying this process.

Ubiquitin possesses seven lysine residues capable of forming different types of linkages. K48- and K63-linked polyubiquitin chains are most abundant linkage types in cells, accounting for approximately half of all ubiquitination events 24, 42. Historically, it was believed that specific types of ubiquitin chains signal substrates for particular forms of degradation. For example, K48-linked polyubiquitin chains were thought to trigger proteasomal degradation, whereas K63-linked chains were associated with lysosomal degradation 43. However, ongoing research has revealed that certain types of linkages can target substrates to multiple degradation pathways. For instance, both K48- and K63-linked ubiquitin chains, can lead to proteasomal as well as lysosomal degradation. Nevertheless, research on the precise mechanisms of K48-linked polyubiquitin chain-mediated lysosomal degradation remains limited. Here, we found that GK promotes GPX4 lysosomal degradation along with an increase in K48-linked ubiquitination, while K63-linked ubiquitination is not involved. Additionally, removing the K48-linked polyubiquitination chain abolished GPX4 lysosomal translocation, indicating the role of K48-linked polyubiquitination in lysosomal degradation.

E3 ubiquitin ligases play a central role in the formation of polyubiquitination chains 44,45 and are critical modulators of cancer development 46. The specific type of ubiquitin chain induced by an E3 ubiquitin ligase can either promote or inhibit the degradation of substrates, thereby influencing ferroptosis. For example, TRIM26 inhibits GPX4 degradation and suppresses ferroptosis by promoting the formation of K63-linked ubiquitin chains 47, whereas TRIM25 induces K48-linked ubiquitin chains promotes GPX4 proteasomal degradation, thereby triggering ferroptosis 29. To identify the E3 ligase responsible for GPX4 K48-linked ubiquitination, we applied IP-MS, which were further confirmed by exogenous and endogenous immunoprecipitation. Intriguingly, the binding between TFEB and TRIM25 was significantly enhanced by GK, suggesting that the interaction between TFEB and TRIM25 is positively correlated with the binding of GPX4 and TRIM25. TRIM25's ubiquitin ligase activity is known to be triggered by conformational changes 48. Therefore, the increased interaction between TFEB and TRIM25 may induce a conformational change in TRIM25, which could further promote its binding to GPX4 and facilitate the K48-linked ubiquitination of GPX4. Furthermore, GK also promotes the expression of TRIM25, which is sharply abolished by TFEB knockout. It has been reported that TRIM25 can degrade 14-3-3, decreasing its binding to TFEB, which then allows TFEB to enter the nucleus and ultimately enhance its activity 49. Our results indicate a positive role for TFEB in GK induced elevation of TRIM25, and decreased association between TFEB and 14-3-3 was also observed in GK treated cells. Thus, GK induced TFEB activation may promote the expression of TRIM25, leading to the dissociation of TFEB from 14-3-3 by reducing the stability of 14-3-3, further activating TFEB in turn.

TRIM25 induced K48-linked ubiquitination could promote proteasome degradation 29, however, its role on lysosomal degradation was not elucidated. Here we further discovered the involvement of TRIM25 induced K48-linked ubiquitination in GPX4 lysosomal degradation. In addition, in the IP-MS data, we also found the decrease in the binding of GPX4 and USP5 in GK treated LUAD cells, which was also confirmed by exogenous and endogenous immunoprecipitation. Since the increased binding of GPX4 to TRIM25 and the decreased binding of GPX4 to USP5 occur simultaneously, this indicates the antagonistic role of USP5 in TRIM25-mediated K48-linked ubiquitination. Considering that the increased binding of GPX4 to TRIM25, the decreased binding of GPX4 to USP5, and the upregulation of GPX4 K48-linked ubiquitination, lysosomal translocation, and degradation are all observed following TFEB activation and attenuated by TFEB knockout, it is plausible that TFEB may mediate TRIM25 to enhance the K48-linked ubiquitination of GPX4, ultimately resulting in its lysosomal degradation.

Ubiquitinated substrates need to be recognized by cargo receptors, such as SQSTM1, NBR1, and OPTN, to bind to these receptors, enter the autophagosome, and subsequently fuse with the lysosome to form autolysosomes for degradation. It has been reported that during TAX1BP1-mediated autophagic degradation of GPX4, TAX1BP1 acts as a key autophagy receptor for GPX4, with GPX4 binding to both TAX1BP1 and SQSTM1. However, in our IP-MS results, we did not observe GPX4 binding to TAX1BP1 or SQSTM1. Additionally, we did not detect any interaction between GPX4 and other cargo receptors, such as NBR1 and OPTN, in our IP-MS data. This suggests that the transfer of ubiquitinated GPX4 to the lysosome induced by GK may not be dependent on the autophagosome. In the case of chaperone-mediated autophagy (CMA), HSC70 recognizes GPX4 and transfers it to the lysosome for degradation. At the basal level, GPX4 binding with HSC70 can occur, but when CMA is induced by specific stimuli, the binding of GPX4 to HSC70 is typically increased, promoting GPX4 lysosomal translocation. Importantly, during this process, HSC70 recognizes GPX4 in its non-ubiquitinated form. In our study, we observed that K48-linked polyubiquitination of GPX4 promotes its lysosomal translocation without an increase in GPX4 binding to HSC70. Although CMA-mediated degradation and TAX1BP1-mediated autophagic degradation of GPX4 occur under specific inducers, the GK induced degradation of GPX4 does not seem to align completely with either of these mechanisms. As research progresses, molecules distinct from classical pathways are being discovered to participate in the regulation of ubiquitinated target translocation to the lysosome. For example, Gαs has been shown to promote the lysosomal translocation of ubiquitinated targets 50. Therefore, delving into the precise mechanism by which K48-linked polyubiquitination promotes GPX4 lysosomal translocation induced by GK will be an intriguing focus for future studies.

In conclusion, our study provides previously unidentified mechanistic insights into GK induced ferroptosis, illustrating for the first time the positive role of TFEB in TRIM25-mediated K48-linked polyubiquitination of GPX4 and its lysosomal degradation, as well as the mechanism by which USP5 impedes GK induced binding of TRIM25 and GPX4. These findings underscore the critical function of TFEB in the GK induced lysosomal degradation of GPX4 and enhance our understanding of TRIM25 and K48-linked polyubiquitination in this process. Furthermore, we reveal for the first time that USP5 and TRIM25 exhibit competitive binding with GPX4 in GK treated LUAD, demonstrating a state of dynamic equilibrium that may be more prone to TRIM25 and GPX4 binding during TFEB activation. However, the binding regions, conformational changes and the mechanisms underlying these competitive interactions and subsequent lysosomal translocation of GPX4 require further elucidation. In addition, the activation on TFEB induced by GK may due to the binding mode that do not induce steric hindrance for TFEB's interaction with DNA or its dimerization; instead, it may stabilize these interactions. However, the key binding site and its impact on TFEB activation require further investigation.

## Supplementary Material

Supplementary figures.
