# Supplementary material for: TFEB promotes Ginkgetin-induced ferroptosis via TRIM25 mediated GPX4 lysosomal degradation in EGFR wide-type lung adenocarcinoma
Source: Theranostics. 2025 Feb 10;15(7):2991–3012. doi: 10.7150/thno.106469 (PMC11898290; doi:10.7150/thno.106469)
Supplement: Supplementary file 1 — Supplementary figures. [file thnov15p2991s1.pdf]

Supplementary data

TFEB promotes GK-induced ferroptosis via TRIM25 mediated GPX4 lysosomal degradation in EGFR wide-type lung adenocarcinoma

Hao-Jie Wang[1,2,3#], Ling-feng Dong[1,2#], Li-Li Ding[3], Xiu-Yuan Miao[1,2], Yu-Wen Zhang[1,2], Li-Ping Zhao[1,2], Li-Hua Yu[1,2], Zhen-Rong Guan[1,2], Ya-Ping Jiang[1,2], Xiao-Qi Tang[1,2], Ya-Xin Yan[1,2], Jian-Shu Lou[1,2,*]

[1]*School of Pharmacy, Hangzhou Normal University, Hangzhou, Zhejiang 311121, China;*
[2]*Key Laboratory of Elemene Class Anti-Cancer Chinese Medicines; Engineering Laboratory of Development and Application of Traditional Chinese Medicines; Collaborative Innovation Center of Traditional Chinese Medicines of Zhejiang Province, Hangzhou Normal University, Hangzhou, Zhejiang 311121, China.*
[3]*Shanghai Key Laboratory of Complex Prescription and MOE Key Laboratory for Standardization of Chinese Medicines, Institute of Chinese Materia Medica, Shanghai University of Traditional Chinese Medicine, Shanghai 201203, China*

[#] *These authors contributed equally* to this work

**Correspondence*: Prof. Jian-Shu Lou, E-mail: jlouab@connect.ust.hk

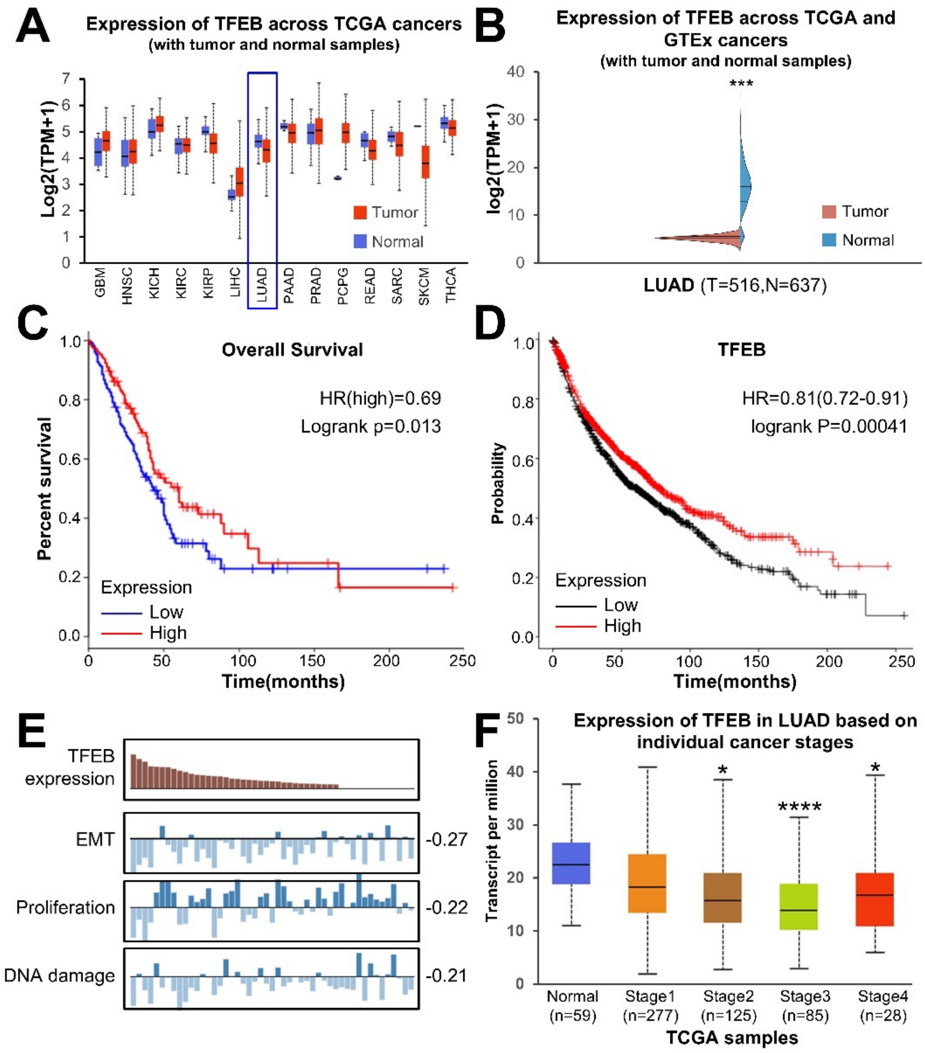

Supplementary Figure S1 TFEB acts as a cancer suppress gene in LUAD. **(A)** Pan-cancer analysis of TFEB expression across various tumor types compared to normal tissues, using data from the TCGA database. **(B)** Pan-cancer analysis of TFEB expression in LUAD tissues compared to normal tissues, integrating data from the TCGA and GTEx databases. **(C, D)** Kaplan–Meier survival analysis of LUAD samples based on TFEB expression levels using the TCGA and GTEx databases (C) and the KM Plotter database (D). **(E)** Functional state analysis of TFEB in LUAD cells from the CancerSEA database. (F) TFEB expression analysis in LUAD tissues at different stages and in normal lung tissues, performed with UALCAN. * *P* < 0.05, **** *P* < 0.001.

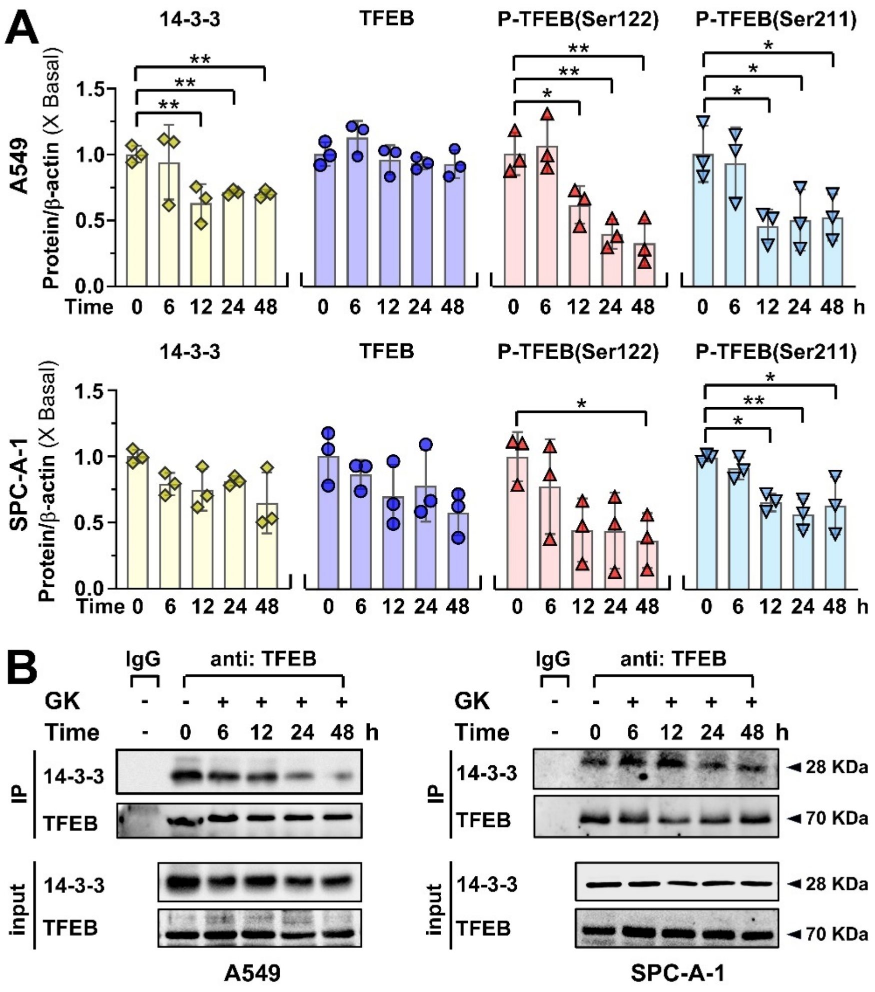

Supplementary Figure S2 The changes of TFEB related protein and the binding of TFEB and 14-3-3 in GK treated LUAD cells.

(A) The semi-quantitative analysis of 14-3-3, TFEB, P-TFEB(Ser122) and P-TFEB(Ser211) expression in figure 1D. *n* = 3, **P* < 0.05, ***P* < 0.01. **(B)** LUAD cells were treated with GK for 6, 12, 24, and 48 h. After treatment, the cells were collected, lysed, and immunoprecipitated using an anti-TFEB antibody. The precipitated proteins or cell lysates were subjected to immunoblotting with anti-14-3-3 and anti-TFEB antibodies to assess protein interactions and expression changes.

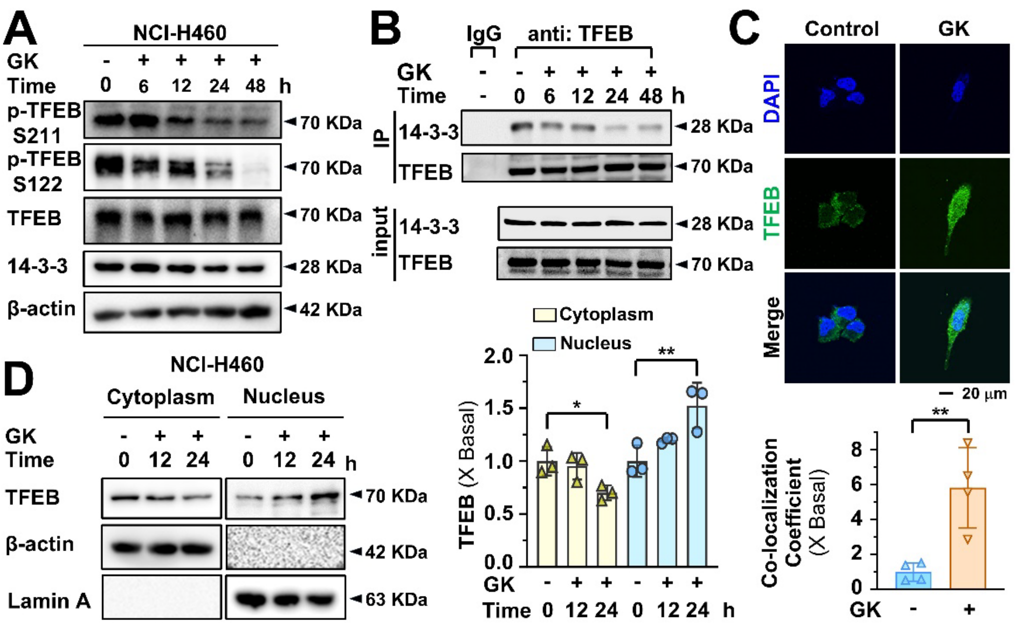

Supplementary Figure S3 GK activates TFEB in LCC cells.

(A) LCC cells NCI-H460 were treated with GK for 6, 12, 24, and 48 h. Western blotting was conducted to analyze the protein levels of TFEB, p-TFEB (Ser122), p-TFEB (Ser211), and 14-3-3. **(B)** LCC cells NCI-H460 were treated as in (A). After treatment, the cells were collected, lysed, and immunoprecipitated using an anti-TFEB antibody. The precipitated proteins or cell lysates were subjected to immunoblotting with anti-14-3-3 and anti-TFEB antibodies to assess protein interactions and expression changes. **(C)** LCC cells NCI-H460 were treated with GK for 24 h, and the nuclear translocation of TFEB was observed by immunofluorescence (upper panel). Scale bar = 20 μm. Co-localization coefficients were calculated by measuring the co-localizing pixels between TFEB (green fluorescence) and DAPI (blue fluorescence) relative to the total number of pixels for the nuclei (DAPI channel) (lower panel). $n = 4$, $^{**}P < 0.01$. **(D)** The cytoplasmic and nuclear protein of LCC cells NCI-H460 were extracted after 24 h GK treatment. The protein expression of TFEB in each fraction were investigated by western blot. β-actin served as a marker of cytoplasm, while Lamin A served as a marker of nucleus (left panel). The semi-quantitative analysis of TFEB protein (right panel). $n = 3$, $^{*}P < 0.05$, $^{**}P < 0.01$.

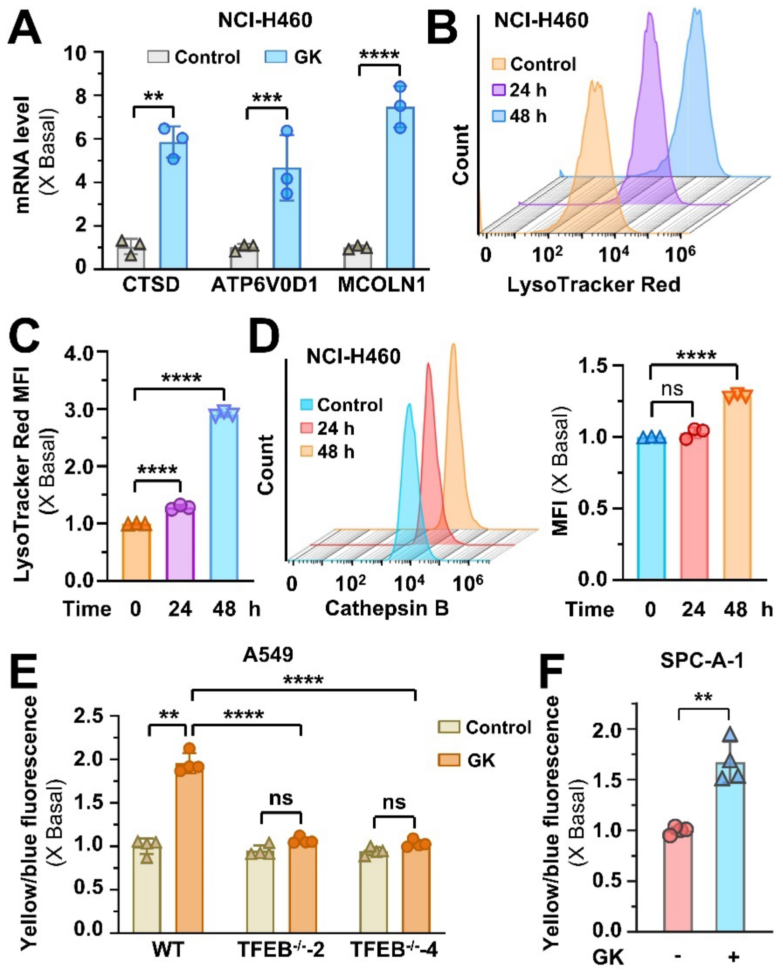

Supplementary Figure S4 GK activates lysosome in LCC cells and decreases lysosome pH in LUAD cells.

(A) LCC cells NCI-H460 were treated with GK (15 μM) for 24 h. Following treatment, cells were harvested, and mRNA was extracted and subsequently reverse transcribed into cDNA. The mRNA level of CTSD, ATP6V0D1 and MCOLN1 were detected by qPCR. n = 3, **P < 0.01, ***P < 0.001, ****P < 0.0001. **(B)** LCC cells NCI-H460 were treated with GK for 24, 48 h, then labeled with LysoTracker™ Red DND-99 (50 nM) for 30 min. Fluorescence intensity of 10,000 cells per sample was measured by flow cytometry. The fluorescence intensity of the cells was displayed in histograms. **(C)** The relative changes in mean fluorescence intensity (MFI) for each treatment group compared to the control group, calculated from (B). n = 3, ****P < 0.0001. **(D)** LCC cells NCI-H460 were treated same as in (B), then stained with Magic Red for 30 min. The level of cathepsin B was analyzed by flow cytometry. Fluorescence intensity of 10,000 cells per sample was analyzed. The fluorescence intensity of the cells

was displayed in histograms (left panel), and the relative changes in mean fluorescence intensity (MFI) compared to the control was quantified (right panel). n = 3, ****P < 0.0001. **(E)** A549 WT cells and TFEB knockout cells (TFEB$^{-/-}$-2, TFEB$^{-/-}$-4) were treated with GK for 24 h, then stained with LysoSensor™ Yellow/Blue DND-160 (1 μM) for 5 min. The blue fluorescence and yellow fluorescence were detected using a Multiskan™ FC Microplate Photometer. The ratio of the intensity of yellow fluorescence relative to blue fluorescence was calculated to indicate the pH in the lysosome. n = 4, **P < 0.01, ****P < 0.0001. **(F)** SPC-A-1 cells were treated with GK and tested the same as in (E), the ratio of the intensity of yellow fluorescence relative to blue fluorescence was calculated. n = 4, **P < 0.01.

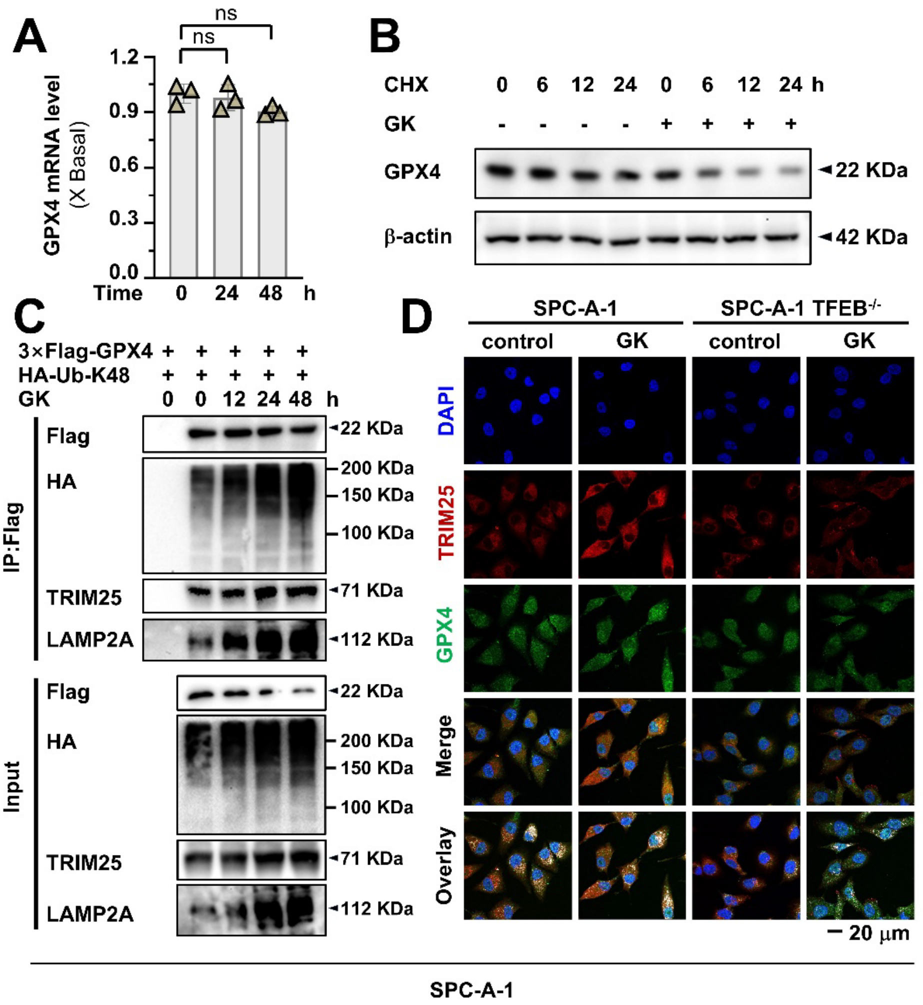

Supplementary Figure S5 GK promoted GPX4 lysosomal degradation and the binding of GPX4 with TRIM25 in SPC-A-1 cells.

(A) SPC-A-1 cells were treated with GK (15 μM) for 24, 48 h. The cells were

harvested, and mRNA was extracted and reverse transcribed into cDNA. The mRNA level of GPX4 was investigated by qPCR. $n = 3$. **(B)** A549 cells were treated with CHX (1 μg/mL), CHX+GK (1 μg/mL + 15 μM) for 6, 12, 24 h, the protein expression of GPX4 was observed by western blot. **(C)** SPC-A-1 cells were transfected with 3×Flag-GPX4 and HA-Ub-K48, then treated with GK for 12, 24, 48 h, cells were collected and lysed. 100 μg of the cell lysates of each sample were subdivided and used as input control. The left cell lysates were subjected to immunoprecipitation via protein G beads and Flag antibody. The expression of K48-linked polyubiquitin, flag-GPX4, TRIM25 and LAMP2A in immunoprecipitants and input were investigated by western blot. **(D)** SPC-A-1 cells and TFEB knockout cells were treated GK (15 μM) for 12 h. The co-localization of GPX4 and TRIM25 was observed by immunofluorescence assay. Scale bar = 20 μM.

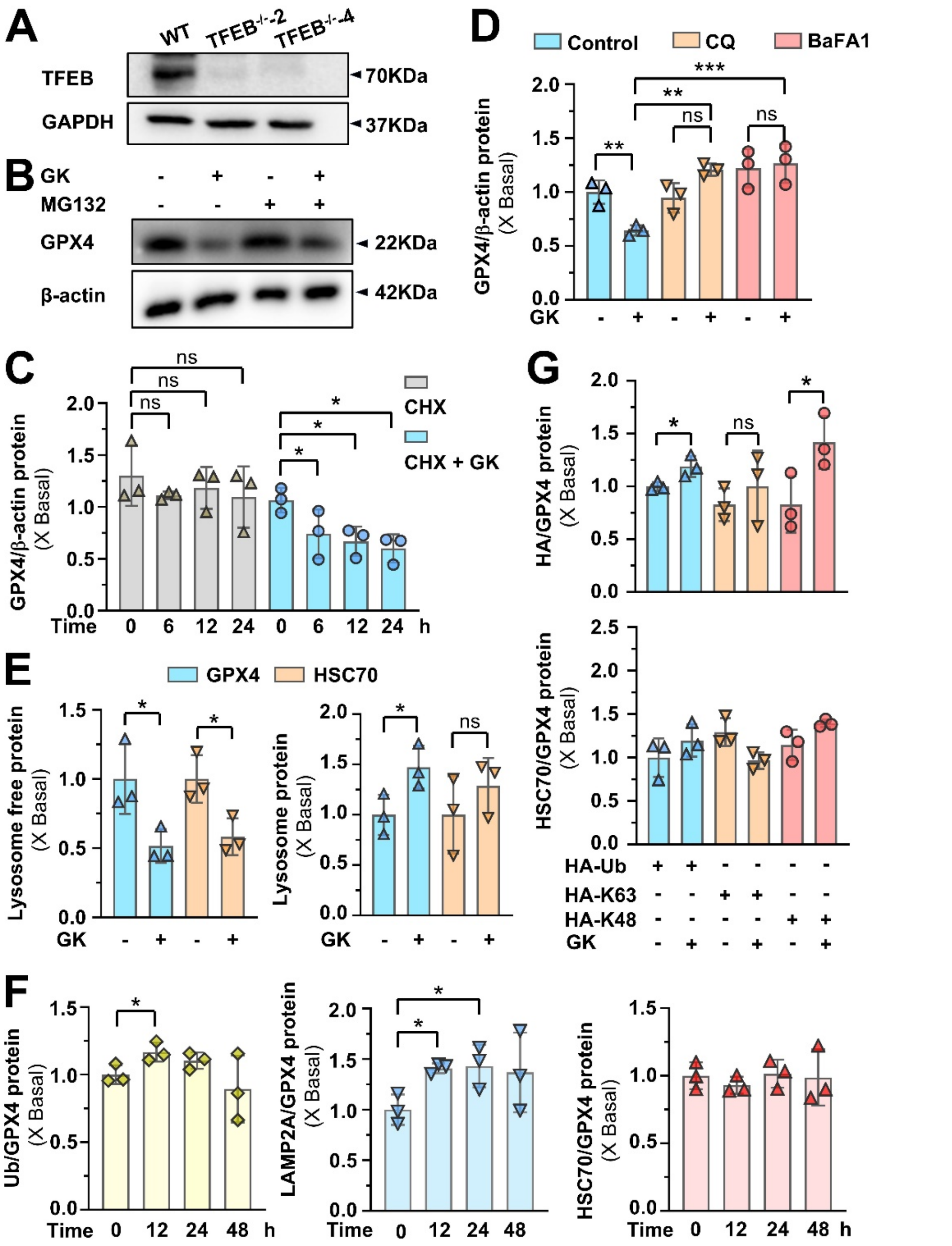

Supplementary Figure S6 Semi-quantitative analysis of protein changes in GK induced degradation of GPX4.

(A) Generation of TFEB knockout cells using the CRISPR-Cas9 system. Single cells were seeded in 96-well plates and cultured for 2–3 weeks to form single-cell colonies. Two monoclonal knockout (KO) cell lines with stable TFEB knockout were selected (A549 TFEB$^{-/-}$-2 and A549 TFEB$^{-/-}$-4). Western blot analysis of TFEB expression in A549 cells and TFEB knockout A549 cells. **(B)** A549 cells were treated with GK in the presence or absence of MG132, the

expression of GPX4 were analyzed by western blot. **(C, D)** Semiquantitative analysis of GPX4 expression from Figure 4B (C) and Figure 4C (D). *n* = 3. *P < 0.05, **P < 0.01, ***P < 0.001. **(E)** Semiquantitative analysis of GPX4 and HSC70 expression in lysosomal and lysosomal fee fractions from Figure 4D. *n* = 3, *P < 0.05. **(F, G)** Semiquantitative analysis of LAMP2A, ubiquitin, and HSC70 from Figure 4G (F), as well as the expression of HA-ubiquitin and HSC70 in the IP fraction from Figure 4H (G). *n* = 3. *P < 0.05.

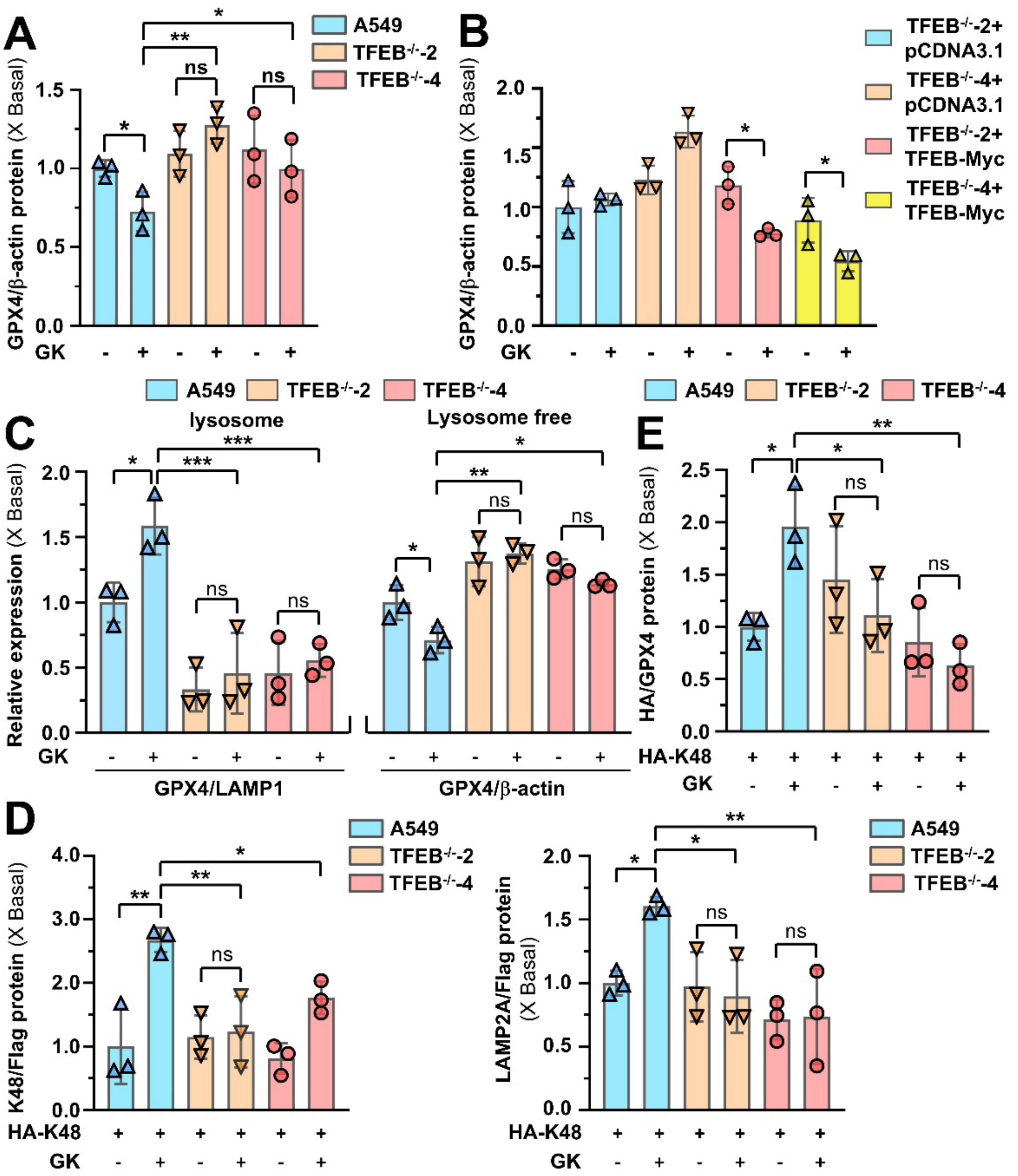

Supplementary Figure S7 Semi-quantitative analysis of protein changes in GK induced K48-linked ubiquitination, lysosome translocation and degradation of GPX4 both in A549 and TFEB knockout A549 cells.

(A, B) Semiquantitative analysis of GPX4 from Figure 5A (A), as well as the

expression of GPX4 from Figure 5B (B). **(C)** Semiquantitative analysis of GPX4 both in lysosome and lysosome free fraction from Figure 5C (C). **(D, E)** Semiquantitative analysis of K48-liked polyubiquitin and LAMP2A in IP fraction from Figure 5F (D), as well as the expression of HA-K48-liked ubiquitin in IP fraction from Figure 5G (E). $n = 3$. *$P < 0.05$, **$P < 0.01$, ***$P < 0.001$.

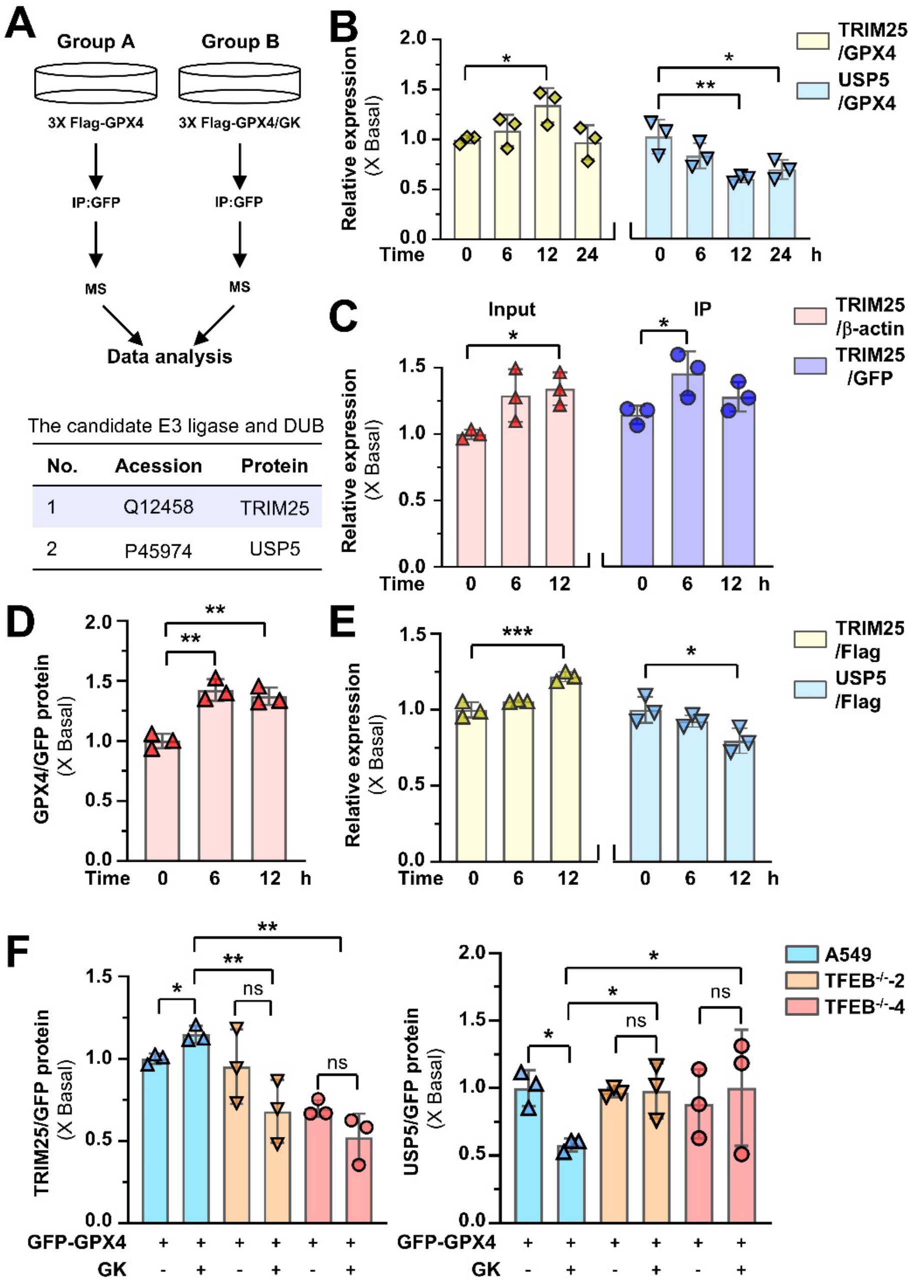

Supplementary Figure S8 Semi-quantitative analysis of protein changes in GK induced increase on the binding of GPX4 and TRIM25 both in A549 and TFEB knockout A549 cells.

(A) GPX4 stable transfection cells were treated or untreated with GK for 24 h.

Following treatment, cells were lysed, and immunoprecipitation was performed. The precipitates were analyzed using LC-MS. TRIM25 and UPS5 are most abundant E3 and DUBs in precipitants respectively. **(B, E)** Semiquantitative analysis of TRIM25 and USP5 expression in IP fraction from Figure 6A (B) and Figure 6D (E). **(C)** Semiquantitative analysis of TRIM25 both in IP and input fractions from Figure 6B. **(D)** Semiquantitative analysis of GPX4 from Figure 6C. **(F)** Semiquantitative analysis of TRIM25 and USP5 expression in the IP fraction of A549 and TFEB knockout A549 cells from Figure 6E. n = 3, *P < 0.05, **P < 0.01, ***P < 0.001.

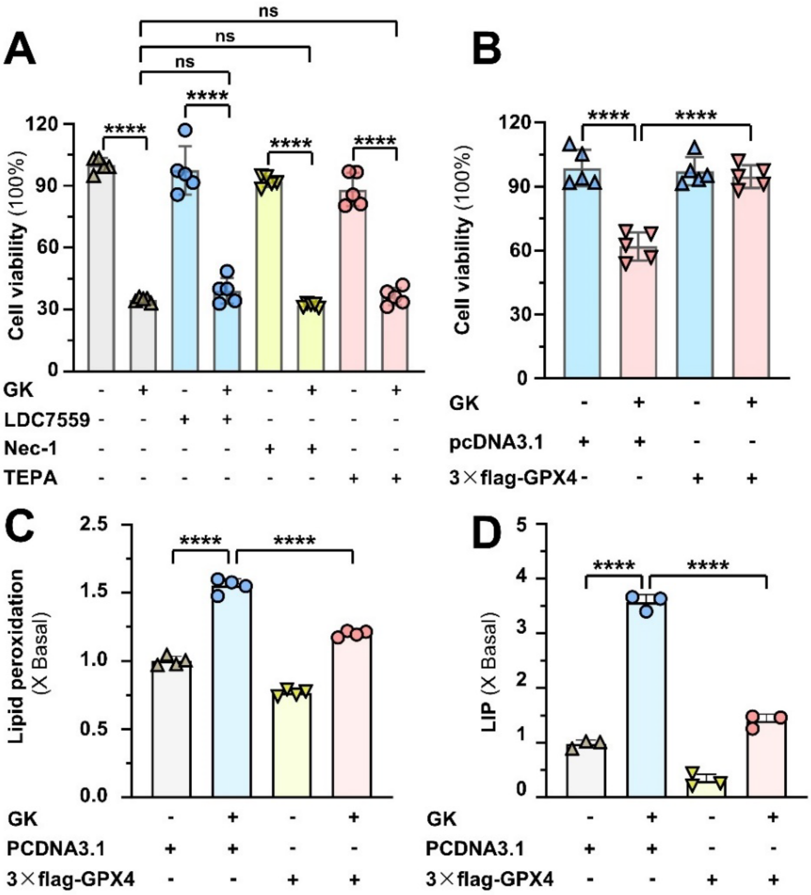

Supplementary Figure S9 GK induced ferroptosis is compromised by GPX4 overexpression

(A) A549 cells were treated with LDC7559 (5 μM), or Necrostatin-1 (10 μM), or TEPA (1 mM) for 1h, then treated with GK for another 48 h. Cell viability was detected by CCK-8 assay. n = 5. **(B)** A549 cells were transfected with 3×flag-GPX4 or mock transfected with pcDNA3.1, then treated with GK for 24 h, cell viability was detected by MTT assay. n = 5. **(C)** The transfection and drug treatment were same as in (B). The cells were collected and stained with

BODIPY™ 581/591 C11 (10 μM) for 30 min. The level of lipid peroxidation was observed by flow cytometry (λexc = 488 nm), 10,000 cells for each sample were analyzed. *n* = 4. **(D)** The transfection and drug treatment were same as in (B). Then the cells were stained with CA-AM (0.5 μM), followed by iron chelator deferiprone (DFP, 100 μM) for 1 h or left untreated. The level of LIP was detected by flow cytometry (λexc = 488 nm), 10,000 cells for each sample were analyzed. *n* = 3. ****$P < 0.0001$.

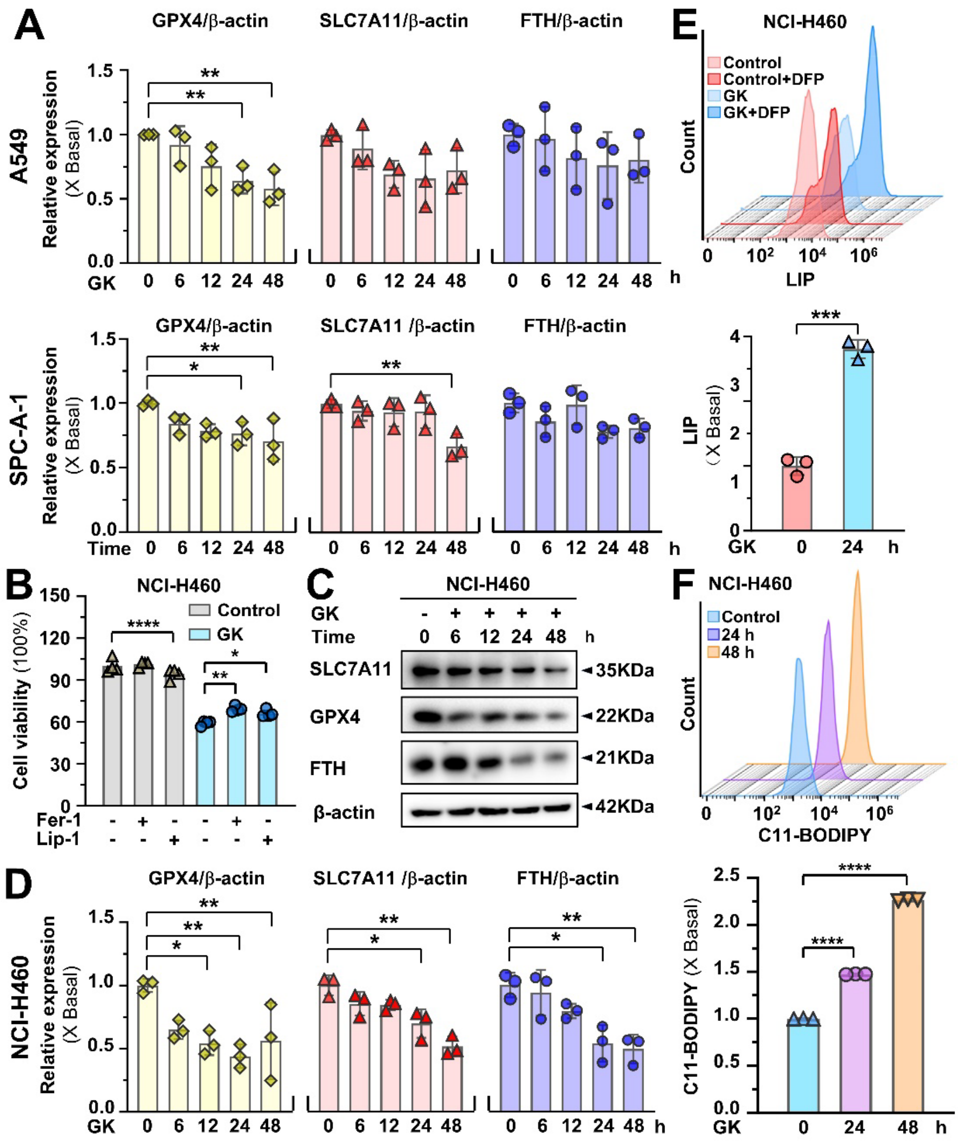

Supplementary Figure S10 GK promote ferroptosis in LCC cells, and the changes of ferroptosis related protein in GK treated LUAD cells.

(A) Semiquantitative analysis of GPX4, SLC7A11 and FTH in GK treated LUAD cells from Figure 7B. *n* = 3, *$P < 0.05$, **$P < 0.01$. **(B)** LCC cells (NCI-H460)

were treated with GK (15 μM) for 48 h in the presence and absence of Ferrostatin-1 or liproxstatin-1. The proliferation inhibition of the cells was observed by MTT assay. n = 4, *P < 0.05, **P < 0.01, ****P < 0.0001. **(C)** LCC cells (NCI-H460) were treated with GK (15 μM) for 6, 12, 24, 48 h. The protein levels of SLC7A11, GPX4, and FTH were observed by western blot. β-actin serves as internal control. **(D)** Semiquantitative analysis of GPX4, SLC7A11 and FTH from (C). n = 3, *P < 0.05, **P < 0.01. **(E-F)** LCC cells (NCI-H460) were treated with GK (15 μM) for 24 h, CA-AM was added to cells at the final concentration of 0.5 μM, followed by adding iron chelator deferiprone (DFP, 100 μM) for 1 h or left untreated. The level of LIP was detected by flow cytometry (λexc = 488 nm) (E). LCC cells (NCI-H460) were treated with GK (15 μM) for 24, 48 h. The cells were collected and stained with BODIPY™ 581/591 C11 for 30 min. The level of lipid peroxidation was observed by flow cytometry (λexc = 488 nm) (F). 10,000 cells for each sample were analyzed. Up panel: The fluorescence intensity of the cells was displayed in histograms. Lower panel: The relative changes in ΔMFI (E) or mean fluorescence intensity (MFI) (F) compared to the control group. n = 3, ***P < 0.001, ****P < 0.0001.
